# Cadherins and growth factor receptors – ligand-selective mechano-switches at cadherin junctions

**DOI:** 10.1242/jcs.262279

**Published:** 2025-02-17

**Authors:** Vinh Vu, Brendan Sullivan, Evan Hebner, Zainab Rahil, Yubo Zou, Deborah Leckband

**Affiliations:** ^1^ Department of Biochemistry, University of Illinois, 600 South Mathews Ave, Urbana, IL 61801, USA; ^2^ Department of Bioengineering, University of Illinois, 1402 W Green St., Urbana, IL 61801, USA; ^3^ Department of Chemical and Biomolecular Engineering, University of Illinois, 600 South Mathews Ave, Urbana, IL 61801, USA; ^4^ Department of Chemistry, University of Illinois, 600 South Mathews Ave, Urbana, IL 61801, USA; ^5^ Carl W. Woese Institute for Genomic Biology, 1206 West Gregory Drive, Urbana, IL 61801, USA

**Keywords:** Cadherin, Force transduction, Growth factor receptor, Heterophilic adhesion

## Abstract

This study investigated possible mechanisms underlying differences between heterophilic and homophilic cadherin adhesions that influence intercellular mechanics and multicellular organization. Results suggest that homophilic cadherin ligation selectively activates force transduction, such that resulting signaling and mechano-transduction amplitudes are independent of cadherin-binding affinities. Epithelial (E-) and neural (N-)cadherin cooperate with distinct growth factors to mechanically activate force transduction cascades. Prior results have demonstrated that E-cadherin and epidermal growth factor receptor form force-sensitive complexes at intercellular junctions. Here, we show that the reconstitution of N-cadherin force transduction requires the co-expression of N-cadherin and fibroblast growth factor receptor. Mechanical measurements further demonstrated that homophilic ligation initiates receptor tyrosine kinase-dependent force transduction cascades, but heterophilic cadherin ligands fail to activate signaling or generate stereotypical mechano-transduction signatures. The all-or-nothing contrast between mechano-transduction by heterophilic versus homophilic cadherin adhesions supersedes differences in cadherin adhesion strength. This mechano-selectivity impacts cell spreading and traction generation on cadherin substrates. Homophilic ligation appears to be a key that selectively unlocks cadherin mechano-transduction. These findings might reconcile the roles of cadherin recognition and cell mechanics in the organization of multicellular assemblies.

## Teaser

**Highlighted Article:** Homotypic, but not heterotypic, cadherin binding cooperates with growth factor receptors to unlock cadherin mechanotransduction cascades and reinforce cell adhesion.

## INTRODUCTION

Cadherins are essential intercellular adhesion proteins that maintain cell–cell cohesion, regulate the integrity of barrier tissues and guide cell segregation in tissue morphogenesis (Gumbiner, 2005; Nose et al., 1988; Takeichi, 1995; Takeichi et al., 1990; Takeichi and Nakagawa, 2001). They are also signaling and force transduction hubs that regulate a range of cell functions (Gumbiner and Kim, 2014; Tzima et al., 2005; Yonemura et al., 2010). The ∼20 different classical cadherin subtypes differ in their primary sequences, and their expression is restricted to distinct tissues (Shapiro and Weis, 2009; Takeichi and Nakagawa, 2001). Both *in vitro* and *in vivo* studies show that cells expressing different cadherin subtypes tend to segregate from each other (Friedlander et al., 1989; Halbleib and Nelson, 2006; Nose et al., 1988; Steinberg, 1963; Tepass et al., 2002). In *Drosophila*, homophilic DE-cadherin adhesion guides oocyte positioning during follicle oogenesis (Godt and Tepass, 1998). In micropipette studies, cells that expressed different cadherins did not bind, even after extended cell–cell contact (Chu et al., 2004). Such observations contributed to the view that cadherins are intrinsically homophilic binding proteins with low heterophilic binding affinities (Halbleib and Nelson, 2006; Koch et al., 1999; Takeichi et al., 1990).

In contrast to what was found in cell-based studies, biophysical measurements have shown that type I classical cadherins form both homophilic and heterophilic bonds, with heterophilic bond energies (affinities) that are intermediate between each of the two homophilic interactions (Katsamba et al., 2009; Niessen and Gumbiner, 2002; Prakasam et al., 2006). The apparent discrepancy between intercellular adhesion and protein level binding strength suggests that additional mechanisms beyond cadherin adhesion energies and expression levels regulate cadherin-selective intercellular adhesion (Chan et al., 2017; Maître et al., 2012; Takeichi, 2023).

Cortical tension is also a major factor influencing multicellular organization (Campas et al., 2023). Protein binding energies cannot account for the tissue surface tension associated with cadherin-dependent cell segregation (Chan et al., 2017; Foty et al., 1996; Krieg et al., 2008; Maître and Heisenberg, 2011; Ninomiya and Winklbauer, 2008). Cortex tension has even been found to supersede cell–cell cohesion in some studies (Krieg et al., 2008). A challenge has been to reconcile the roles of cadherin recognition, cell–cell cohesion and cell mechanics in multicellular organization.

Cadherin mechano-transduction could be the link between cadherin specificity, differential cell adhesion and cell mechanics. Cadherin force transduction activates contractility and cytoskeletal reinforcement at intercellular adhesions (Leckband and de Rooij, 2014). The cytosolic protein α-catenin couples cadherins to filamentous actin, and undergoes a force-dependent conformational change that supports local cytoskeletal remodeling at cell adhesions (Barry et al., 2014; Buckley et al., 2014; Kim et al., 2015; Yao et al., 2014; Yonemura et al., 2010). However, α-catenin unfurls under nonspecific tension (Yao et al., 2014), and there is no evidence of cadherin-selective activation.

By contrast, a second force transduction mechanism activates signaling in a cadherin (ligand)-selective manner (Sullivan et al., 2022; Tabdili et al., 2012b). Co-immunoprecipitation and fluorescence resonance energy transfer (FRET) showed that epithelial E-cadherin (E-cad, encoded by *CDH1*) binds epidermal growth factor receptor (EGFR, ErbB1) at the plasma membranes of live cells (Qian et al., 2004; Leckband, 2023; Sehgal et al., 2018; Sullivan et al., 2022). Junctional tension mechanically disrupts the hetero-receptor complex to initiate EGF-dependent cascades that regulate junction remodeling, cell contractility and proliferation (Sullivan et al., 2022). VE-cadherin similarly cooperates with VEGFR2 and VEGFR3 to regulate endothelial shear alignment (Coon et al., 2015), and stretching endothelial monolayers disrupts VE-cadherin complexes with VEGFR2 to initiate signaling (Kong et al., 2022; Tian et al., 2016). However, E-cad force transduction is not activated by heterophilic (N-cadherin; N-cad, encoded by *CDH2*) ligand or by tugging with anti-E-cad antibodies. Likewise, N-cad force transduction is not activated by E-cad ligand (Tabdili et al., 2012b). Mechanically perturbing VE-cadherin with anti-VE-cadherin antibodies also fails to trigger force transduction (Barry et al., 2015).

Mechano-transduction appears to require both tension and specific cadherin ligation (Barry et al., 2015; Tabdili et al., 2012b). This selectivity is not explained by cadherin-binding affinities. Although heterophilic E-cad–N-cad bonds are stronger than homophilic N-cad bonds (Katsamba et al., 2009; Niessen and Gumbiner, 2002; Prakasam et al., 2006), E-cad ligand does not activate N-cad force transduction (Tabdili et al., 2012b). The amplitudes of ligand-dependent force transduction responses do not scale with differences in binding affinities. Force alone is also not sufficient; namely, the activation of VE-cadherin mechano-transduction using antibody-modified beads depends on the VE-cadherin epitope targeted (Barry et al., 2015).

Here, we tested the hypothesis that homophilic and heterophilic cadherin bonds have starkly different capacities to activate mechano-transduction, despite robust protein-level heterophilic binding. The results provide new insight into the role of cadherin selectivity, force transduction signaling and its regulation by receptor tyrosine kinases and cadherin ligation. Studies further investigated the impact of this mechano-selectivity on downstream signaling and associated mechanical functions of cell attachment, spreading and traction generation.

## RESULTS

### Quantification of relative cadherin adhesion strengths

**Fig. 1.**
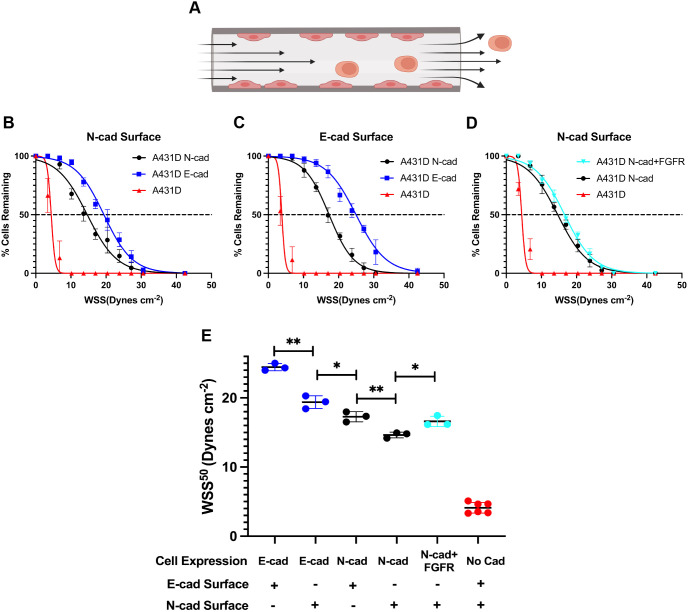
**Heterophilic and homophilic cadherin bonds support cell adhesion.** (A) Schematic of the capillary shear flow assay. Created in BioRender by Leckband, D., 2025. https://BioRender.com/n70i793. Republished with permission. (B–D) Percentage of cells retained in cadherin-coated capillaries versus the wall shear stress (WSS, dynes cm^−2^). Solid curves are non-linear least squares fits of the data to Eqn 1, with best fit parameters given in the text and summarized in part E. The dashed, horizontal lines denote 50% of cells remaining. (B) Cells expressing N-cad (A431D^N-cad^), E-cad (A431D^E-cad^) or no cadherin (A431-D) adhered to N-cad modified capillaries. (C) Cells expressing N-cad (A431D^N-cad^), E-cad (A431D^E-cad^) or no cadherin (A431-D) adhered to E-cad modified capillaries. In controls, A431D^E-cad^ cells were treated with EGTA. (D) A431-D cells expressing exogenous N-cad and FGFR1 (A431D^N-cad+FGFR^), N-cad only (A431D^N-cad^) or no cadherin (A431-D) adhered to N-cad modified capillaries. (E) Graphs of quantified WSS^50^ values under conditions indicated in B–D. Data indicate the mean±s.d. *N*_exp_=3 for each. *P<0.05; **P<0.005 (unpaired two-tailed *t*-test).

To verify that differences in force transduction are not caused solely by differences in cadherin bond strength, the relative strength of cadherin-mediated adhesion was quantified using capillary flow assays (Fig. 1A). Studies used A431-D epithelial cells engineered to stably express either E-cad (A431-D^E-cad^) or N-cad (A431-D^N-cad^). Engineered cells used in all these studies were sorted for similar surface expression using quantitative flow cytometry (Chien et al., 2008; Tabdili et al., 2012a). The quantified average surface densities of E-cad and N-cad on A431-D cells were ∼25 and ∼28 cadherins/µm^2^, respectively. The cells were seeded on the inner walls of glass capillaries that were coated with physisorbed Fc-tagged extracellular domains of N-cad (N-cad-Fc) or E-cad (E-cad-Fc). To prevent spreading, so that adhesion reflected differences in protein bond strengths, cells were seeded in low-serum (0.5%) medium.

The quantified percentages of A431-D^N-cad^ versus A431-D^E-cad^ cells retained on N-cad-Fc coated capillaries, as a function of the wall shear stress (WSS), are plotted in Fig. 1B. We compared the wall shear stresses at which 50% of the cells were retained, WSS^50^, based on data fits to Eqn 1 (Garcia et al., 1997)
(1)

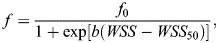
which models cell detachment under shear stress. Here, *f* is the experimentally determine fraction of adherent cells and *f*_0_, *b* and *WSS*_50_ are the fitted zero-stress fraction of bound cells (∼1.0), the decay slope, and the shear stress at which 50% of the cells are retained (inflection point). The thus fitted WSS_50_ values (MatLab) are summarized in Fig. 1E. Fig. 1C compares the adhesion of A431-D^N-cad^ and A431-D^E-cad^ cells on E-cad-Fc-coated capillaries. A431-D^E-cad^ cells adhered more robustly to E-cad-Fc than A431-D^N-cad^ cells did (Fig. 1E), and in both cases adhesion was stronger than control cells without cadherin. The relative pairwise cadherin adhesion strengths are E-cad–E-cad>E-cad–N-cad>N-cad–N-cad. Thus, heterophilic adhesion is intermediate between the two homophilic bonds, in agreement with prior reports (Niessen and Gumbiner, 2002; Prakasam et al., 2006; Katsamba et al., 2009).

FGFR expression slightly increased the homophilic N-cad adhesion. The A431-D^N-cad^ and A431-D^N-cad+FGFR^ cells express similar N-cad levels (∼25–28 cadherins/µm^2^), but their WSS^50^ values differed slightly (Fig. 1D). The WSS^50^ for A431-D^N-cad+FGFR^ and A431-D^N-cad^ cells on N-cad-Fc were 17±0.7 dynes/cm^2^ and 15±0.4 dynes/cm^2^ (mean±s.d.), respectively (Fig. 1E).

### E-cad force transduction requires homophilic ligation and EGFR activity

**Fig. 2.**
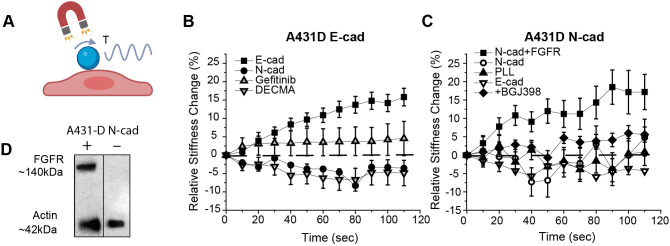
**MTC measurements reveal significant differences in force transduction by homophilic and heterophilic cadherin adhesions.** (A) Schematic of the MTC setup. Ligand-coated ferromagnetic beads adhere to the apical surface of A431-D cells expressing either human E-cad or N-cad. The magnetized beads are subjected to an oscillating orthogonal magnetic field, which generates an oscillating torque (*T*) that displaces the beads. Created in BioRender by Leckband, D., 2025. https://BioRender.com/o99i138. Republished with permission. (B) Relative stiffness change (%) during 2 min of loading beads bound to A431-D^E-cad^ cells. Beads were coated with E-cad (black squares), N-cad (black circles) or DECMA-1 (anti-E-cad antibody, inverted triangle). In controls with E-cad beads, cells were treated with Gefitinib (white triangle). Number of beads per condition: *n*_E-cad_=259, *n*_N-cad_=168, *n*_E-cad+Gefitinib_=226, *n*_E-cad+DECMA1_=136. *N*_exp_=3. (C) Relative stiffness change (%) during 2 min of loading beads on A431D^N-cad^ cells with or without FGFR co-expression, as indicated in the figure. Beads were coated with N-cad (black squares), E-cad (inverted white triangles), or PLL (black triangles). A431D^N-cad^ cells (without FGFR1) were probed with N-cad beads (white circles, −FGFR). A431D^N-cad+FGFR^ cells probed with N-cad beads were treated with the FGFR inhibitor BGJ398 (black diamonds). Number of beads per condition: *n*_N-cad+FGFR_=200, *n*_N-cad (–FGFR)_=150, *n*_PLL_=148, *n*_E-cad_=298, *n*_BGJ398_=300. *N*_exp_=3. (D) Western blots of FGFR1 in A431D^N-cad^ cells transfected with FGFR1 (+, left) and non-transfected A431D^N-cad^ cells (−, right). Number of western blots=2. Data in B and C are the mean±s.d.

Studies next tested whether the relative adhesion strengths proportionally activate force transduction. Here, magnetic twisting cytometry (MTC) (Wang et al., 1993) quantified cell responses to tugging on cadherin receptors with ferromagnetic beads coated with cadherin extracellular domains (Fig. 2A). MTC measurements quantify the viscoelasticity of bead cell junctions (Fig. 2A), in response to an oscillating field (*H*), which generates a twisting torque (*T*) on the beads. Cadherin force transduction triggers an increase in the junction modulus (cell stiffening) (le Duc et al., 2010).

Fig. 2B shows the measured stiffening response to tugging on E-cad receptors on A431-D^E-cad^ cells, with beads coated with E-cad-Fc, N-cad-Fc or the anti-E-cad antibody DECMA-1. Twisting homophilic bonds between A431-D^E-cad^ cells and E-cad-Fc-coated beads (E-cad beads) triggered a 16±2% (mean±s.d.), relative increase in cell stiffness (Fig. 2B). However, N-cad-beads, when bound to A431-D^E-cad^ cells did not trigger a stiffening response (−3.6±0.3%), despite robust heterophilic N-cad–E-cad adhesion (Fig. 1B,E). In controls with DECMA-1-coated beads, the relative cell change was −4.9±3.4%.

Prior studies have implicated EGFR in E-cad force transduction (Sullivan et al., 2022). We found that the non-competitive EGFR kinase inhibitor Gefitinib reduced A431-D^E-cad^ cell stiffening activated by E-cad beads to 4.5±5% (Fig. 2B). This agrees with a prior report showing that knocking down EGFR expression or EGFR inhibition suppresses E-cad force transduction (Muhamed et al., 2016; Sehgal et al., 2018; Sullivan et al., 2022). MCF-7 cells, which express endogenous human E-cad and normal EGFR levels behaved similarly. Twisting E-cad beads on MCF-7 cells triggered a 17±5% increase in stiffness (Fig. S1A). However, beads coated with N-cad-Fc or with poly(L-lysine) (PLL) failed to activate cell stiffening, even though N-cad binds E-cad on MCF-7 cells (Fig. S1A; Tabdili et al., 2012b). Controls did not use Fc-coated beads, because they do not bind the cells. Gefitinib abolished E-cad-mediated MCF-7 stiffening (Fig. S1A). The results support the hypothesis that E-cad force transduction requires EGFR activity and that homophilic E-cad ligation is required to activate force transduction by E-cad.

### Co-expression of FGFR1 and N-cad reconstitutes N-cad-mediated force transduction

Studies next tested whether N-cad similarly activated force transduction when expressed in A431-D cells. Surprisingly, tugging on homophilic bonds between N-cad beads and A431-D cells expressing N-cad (A431-D^N-cad^) did not activate cell stiffening, even though the N-cad expression at ∼25 cadherins µm^−2^ was similar to E-cad levels on A431-D^E-cad^ cells (∼28 cadherins µm^−2^). Neither N-cad-Fc beads (4±4%; mean±s.d.) nor E-cad beads triggered cell stiffening (−1±5%, Fig. 2C). This behavior contrasts with C2C12 cells, which stiffened significantly, when perturbed with N-cad beads (Fig. S1B).

We reasoned that N-cad force transduction might involve a different receptor tyrosine kinase than EGFR, which is overexpressed in A431-D cells (Lewis et al., 1997). N-cad crosstalk with fibroblast growth factor receptor (FGFR) regulates cell migration and homophilic adhesion (Nguyen et al., 2019; Nguyen and Mege, 2016; Suyama et al., 2002; Sanchez-Heras et al., 2006; Williams et al., 2001). Also, the EC4 domain of N-cad reportedly binds the acid box region of FGFR to activate the signaling (Suyama et al., 2002; Williams et al., 2001).

Studies tested whether FGFR expression potentiates N-cad force transduction. Western blots confirmed that A431-D cells express negligible FGFR (Fig. 2D). A431-D^N-cad^ cells were then stably transfected with FGFR1 (A431-D^N-cad+FGFR^), as confirmed by western blotting (Fig. 2D). In subsequent MTC measurements with A431-D^N-cad+FGFR^ cells, tugging with N-cad beads increased the cell stiffness by 17±4%, and the FGFR inhibitor BGJ398 abolished the response (Fig. 2C). Pretreating C2C12 cells with the FGFR inhibitor similarly abolished cell stiffening (Fig. S1B). Like C2C12 cells (Fig. S1B) (Tabdili et al., 2012b), heterophilic bonds between E-cad beads and A431-D^N-cad+FGFR^ cells did not activate stiffening (Fig. 2C), despite our finding that N-cad–E-cad adhesion is stronger than N-cad–N-cad adhesion (Fig. 1E). This ligand selectivity is similar to that reported with MBA-MD-435 and C2C12 cells (Fig. S1B), which both express N-cad (Tabdili et al., 2012b). The results show that the co-expression of N-cad and FGFR reconstitutes N-cad force transduction, but force-activated signaling requires homophilic ligation.

### Ligand-dependent spreading and attachment densities are independent of cadherin adhesion strength

Cell spreading is a mechanically activated process. On extracellular matrix, spreading proceeds through biochemical events that are activated by successive mechanical changes in membrane tension, actin flow and cell contractility (Wolfenson et al., 2014). There are parallels between the actin clutch model used to describe integrin-mediated spreading (Chan and Odde, 2008; Elosegui-Artola et al., 2016) and intercellular adhesion (Noordstra et al., 2023). Here, we used measurements of spreading on cadherin-modified substrates to test the hypothesis that force transduction requires cadherin-selective receptor tyrosine kinase (RTK) activity and homophilic ligation. E-cad-mediated force transduction requires EGF (Sehgal et al., 2018; Sullivan et al., 2022), and serum-starved cells do not attach well or spread on cadherin substrates. Thus, measurements were done with low serum (0.5%), to stabilize cell attachment. The fibronectin (FN)-blocking 16G3 antibody was included to block integrin interference caused by secreted FN (Nagai et al., 1991). The influence of RTK activation was determined by comparing spreading with and without added 100 ng ml^−1^ EGF or 100 ng ml^−1^ FGF. Because EGFR activation can trigger E-cad phosphorylation and endocytosis, which reduces adhesion (Lilien and Balsamo, 2005), initial adhesion measurements at different growth factor concentrations were used to establish that 100 ng ml^−1^ EGF in 0.5% serum medium did not reduce adhesion on the measurement time scale of 90 min.

**Fig. 3.**
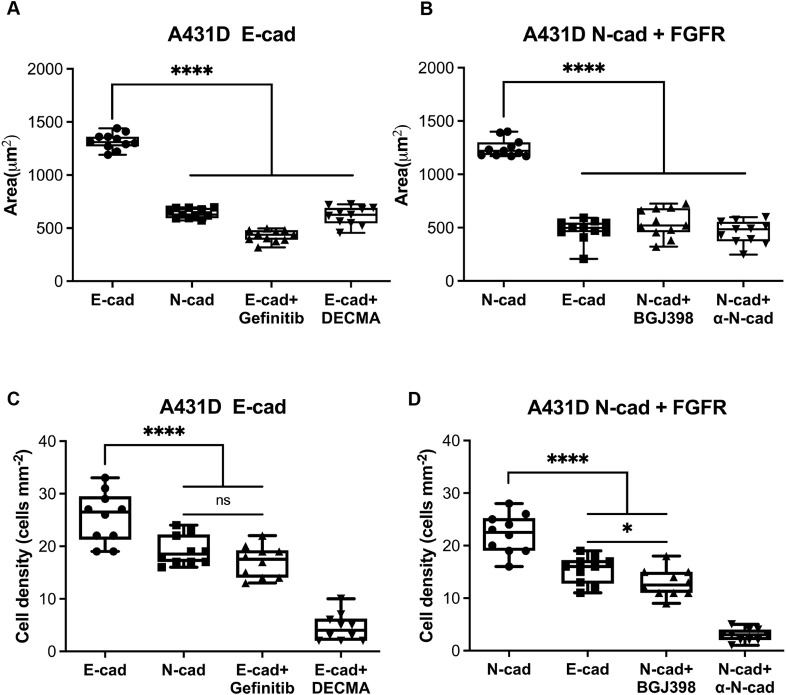
**Ligand-dependent spread cell areas and attached densities on N-cad and E-cad coated polyacrylamide gels.** (A) Average spread area (µm^2^) of A431-D^E-cad^ cells on PAA gels coated with E-cad or N-cad. Cells were treated with EGFR inhibitor (Gefinitib) or with blocking antibody (DECMA). Number of cells per condition: *n*=11; *N*_exp_=2. (B) Average spread area (µm^2^) of A431-D cells co-expressing N-cad and FGFR seeded on polyacrylamide gels coated with E-cad or N-cad. Cells were treated with FGFR inhibitor (BGJ398) or blocking antibody (Anti N-cad). The number of cells per condition: *n*=11; *N*_exp_=2. (C) Average number of A431-D^E-cad^ cells retained per mm^2^ on E-cad or N-cad substrates, 90 min after seeding in low-serum medium. Cell treatments were Gefitinib or DECMA-1). (D) Average number of A431-D^N-cad+FGFR^ cells per mm^2^ on N-cad or E-cad-coated substrates, 90 min after seeding in low serum medium. Cell treatments were BGJ398 or anti-N-cad antibody. For all panels, the first line of the *x*-axis label indicates the cadherin coating, and the second line indicates cell treatments. For C and D, the number of images (ROI=600 µm^2^) per substrate *n*_ROI_=3, *N*_exp_=2. For box plots, the box represents the 25–75th percentiles, and the median is indicated. The whiskers show the complete range. *P<0.05; ****P<0.0001; ns, not significant (unpaired two-tailed *t*-test for 1:1 comparisons; one-way ANOVA for multiple comparisons).

To identify ligand-dependent spreading differences, measurements determined the rate of spreading on different cadherin-coated glass substrates, in the presence and absence of added growth factor. Fig. S2A,B shows the cell areas on, respectively, E-cad- or N-cad-coated glass as a function of time. In low-serum medium, the final spread areas of A431-D^E-cad^ cells on homophilic E-cad substrates with added EGF or A431-D^N-cad+FGFR^ cells on N-cad substrates with added FGF plateaued at ∼90 min (Fig. S2A,B). Without added growth factor, cells initially spread more slowly but eventually reached the same final areas as cells with added growth factor (Fig. S2A,B). On E-cad surfaces, the final areas of A431-D^E-cad^ cells, with and without added EGF were statistically similar at, respectively, 1250±150 and 1224±17 µm^2^ (mean±s.d.) (Fig. 3A; Fig. S2A). Likewise, on N-cad substrates with added FGF, A431-D^N-cad+FGFR^ cells spread to 860±80 µm^2^ (Fig. S2B). Although cells spread more slowly without growth factor, the final areas were similar. Serum-starved cells did not spread on cadherin surfaces (data not shown).

To determine whether cadherin selectivity affected the spreading behavior, similar measurements quantified cell spreading in low serum on glass coated with heterophilic ligand. Although E-cad binds N-cad, the spread area of A431-D^E-cad^ on N-cad surfaces (541±40 µm^2^) was significantly lower than that on E-cad substrates (1320±80 µm^2^) (Fig. 3A) but similar to what was seen with Gefitinib (430±50 µm^2^)- or DECMA-1 (620±90 µm^2^)-treated A431-D^E-cad^ cells on E-cad (Fig. 3A). Similarly, compared to spread areas on N-cad substrates (1250±80 µm^2^), A431-D^N-cad+FGFR^ cells spread less on E-cad (477±104 µm^2^) (Fig. 3B), despite stronger heterophilic N-cad–E-cad versus N-cad–N-cad adhesion (Fig. 1). The average A431-D^N-cad+FGFR^ area on E-cad surfaces was similar to BGJ398-treated cells on N-cad (540±130 µm^2^) (Fig. 3B). These results demonstrate selective cell spreading on homophilic substrates, such that the final areas do not scale with the cadherin affinity. The greater spreading on homophilic surfaces also requires RTK activity.

To verify the generality of ligand-selective spreading and to visualize actin organization in cells on different substrates, additional studies used MCF-7 cells. On E-cad-coated glass in low-serum medium, cells were well spread and exhibited a ‘fried egg’ morphology, with well-defined cortical actin fibers and few filopodia at the edges. Representative images are in Fig. S2E. In contrast, spreading was visibly different on N-cad substrates. Cells did not spread uniformly, and they exhibited prominent filopodia at the periphery. MCF-7 cells treated with DECMA-1, which blocks E-cad adhesion, remained small and rounded, with numerous filopodia at the periphery.

A431-D^E-cad^ cells behaved in a similar manner to MCF-7 cells (Fig. S2E); on E-cad substrates, cells were circular and well spread, with dense actin fibers at the cortex. The cells spread somewhat on N-cad substrates, due to heterophilic binding (Fig. 1E), but they extended large irregular protrusions with filopodia at the tips. DECMA-1-treated A431-D^E-cad^ cells on E-cad substrates remained small and round and exhibited numerous filopodia (Fig. S2E).

The relative densities of attached (retained) cells (number of cells per µm^2^) can also reflect differences in adhesion-activated processes that stabilize attachment (Rahil et al., 2016; Tabdili et al., 2012b). We therefore tested whether ligand-dependent signals effected preferential attachment on homophilic substrates. Fig. 3C shows that statistically more A431-D^E-cad^ cells were retained on E-cad (25±4 cells per µm^2^; mean±s.d.) than on N-cad substrates (19±3 cells per µm^2^) in 0.5% serum, when seeded at similar densities. Cell retention on N-cad was statistically similar to Gefitinib-treated cells on E-cad (17±3 cells per µm^2^) (Fig. 3C). DECMA-1 further reduced the cell density (4.5±2.6 cells per µm^2^), confirming E-cad-mediated binding.

Similarly, more A431-D^N-cad+FGFR^ cells attached to homophilic N-cad (22±4 cells per µm^2^) than to E-cad substrates (15±3 cells per µm^2^) (Fig. 3D). Treatment with the FGFR inhibitor BGJ398 reduced A431-D^N-cad+FGFR^ cell densities on N-cad surfaces (13±3 cells per µm^2^) to similar levels as those seen for untreated cells on E-cad (Fig. 3D). Pre-treatment with anti-N-cad antibody further reduced A431-D^N-cad+FGFR^ attachment densities (2.9±1.1 cells per µm^2^), confirming N-cad-mediated binding (Fig. 3D).

### Traction forces on cadherin substrates depend on the cadherin ligand and RTK activity

**Fig. 4.**
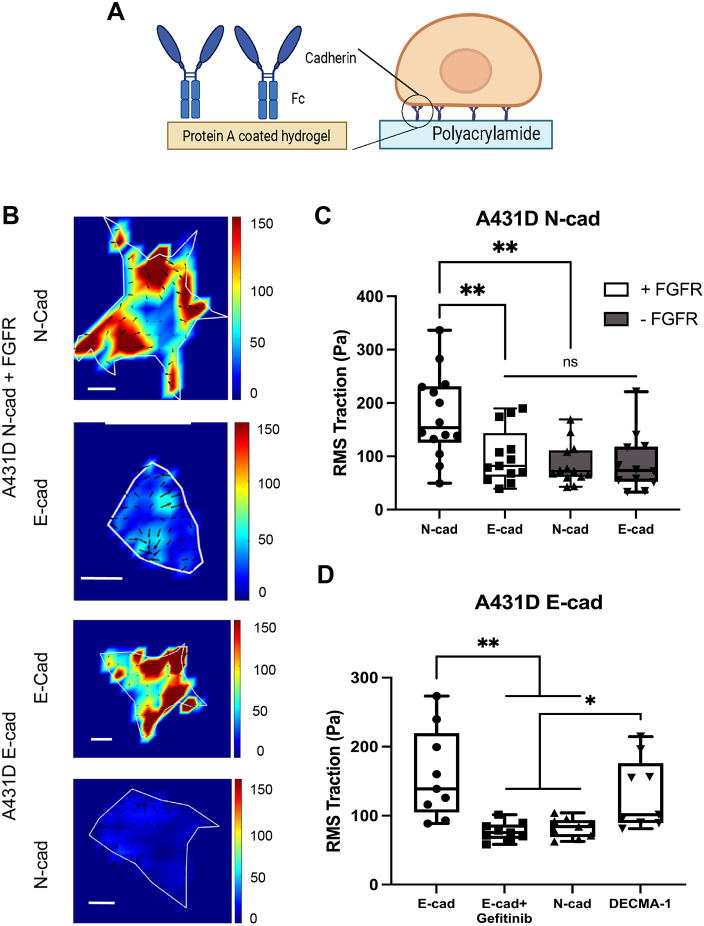
**Traction forces depend on the immobilized cadherin ligand and RTK activity.** (A) Substrate configuration used in traction force measurements. Cells were seeded on polyacrylamide gels (40 kPa) that were covalently modified with Protein A, which captures and orients Fc-tagged cadherin ectodomains or DECMA-1 antibody. Cells were cultured in low-serum (0.5%) medium. Created in BioRender, Leckband, D., 2025. https://BioRender.com/k01m454. Republished with permission. (B) Heat maps of the constrained traction stress (Pa) generated by single cells on functionalized 40 kPa PAA gels. The upper two images were obtained with A431-D^N-cad+FGFR^ cells on N-cad-Fc or E-cad-Fc modified gels. The bottom two images show traction heat maps generated by A431-D^E-cad^ cells on E-cad gels or N-cad gels. (C) Root mean square (RMS) traction stress (Pa) generated by A431-D^Ncad^ cells on PAA gels modified with E-cad-Fc or N-cad-Fc. Data show A431D^Ncad^ cells with (+FGFR) and without FGFR (−FGFR) expression. Number of cells per condition, *n*=13. Experimental replicates *N*_exp_=2 for each condition. (D) RMS traction stress (Pa) generated by A431-D^Ecad^ cells on 40 kPa gels modified with E-cad, N-cad, or DECMA-1 antibody. Data include Gefitinib treated A431D^Ecad^ cells on E-cad gels with *n*_cells_ (+Gefitinib)=*n*_cells_ (−Gefitinib)=8. *n*_cells_=9 for other conditions. *N*_exp_=2 for all conditions. For box plots, the box represents the 25–75th percentiles, and the median is indicated. The whiskers show the complete range. *P<0.05; **P<0.005; ns, not significant (unpaired two-tailed *t*-test for 1:1 comparisons; one-way ANOVA for multiple comparisons).

Spreading cells generate traction forces through adhesion receptors. To determine whether cadherin mechano-selectivity influences cell tractions, traction force microscopy (TFM) measurements were done with cells on elastomeric polyacrylamide (PAA) gels modified with N-cad-Fc or E-cad-Fc proteins. The gels had elastic moduli of 40 kPa. This modulus is close to the 34 kPa value used previously (Muhamed et al., 2016), and the latter condition provided good signal-to-noise ratios. Fig. 4A illustrates the experimental configuration. Like in spreading measurements, TFM studies used low serum (0.5%) medium, to minimize integrin interference and to ensure low basal RTK activity and sensitivity to added growth factor.

Measurements with N-cad-expressing cells tested the influence of FGFR1 expression and ligand identity on traction generation. To determine the influence of FGFR1, A431-D^N-cad^ cells were compared with A431-D^N-cad+FGFR^ cells. Fig. S3A and Fig. 4B show representative heat maps of strain fields exerted by the cells. FGFR1 co-expression with N-cad significantly increased the root mean square (RMS) traction, relative to cells lacking FGFR (Fig. 4C; Fig. S3B). The dependence of traction amplitudes on the substrate ligand was then determined by comparing A431-D^N-cad+FGFR^ cell tractions on N-cad- versus E-cad-coated gels. The RMS tractions of A431-D^N-cad+FGFR^ cells were significantly larger on homophilic N-cad (176±79 Pa; mean±s.d.) than on E-cad gels (100±51 Pa) (mean±s.d.; Fig. 4C, Fig. S3B). In agreement with MTC and cell spreading data, the ligand selectivity of traction generation was independent of the cadherin binding strength; namely, N-cad bonds were weaker, but they supported significantly higher traction forces than heterophilic adhesions (Fig. S3B).

We further tested how FGFR activation by FGF affected ligand-dependent traction. A431D^N-cad^ cells, which lack FGFR, exerted statistically similar RMS tractions on N-cad (85±38 Pa) and on E-cad substrates (88±54 Pa), and the latter values were similar to those for A431-D^N-cad+FGFR^ cells on E-cad (100±51 Pa) (Fig. 4C; Fig. S3B). In low-serum medium, FGFR1 co-expression with N-cad alone enhanced the contrast between traction forces on N-cad versus E-cad substrates (Fig. 4C). Adding FGF or 10% serum further increased A431-D^N-cad+FGFR^ tractions on N-cad gels, but it had no effect on cells on E-cad substrates (Fig. S3B). Conversely, traction forces of A431D^N-cad^ cells, which lack FGFR, were insensitive to substrate ligand or added FGF (Fig. 4C; Fig. S3B). These results suggest that FGFR switches on force transduction signaling at homophilic adhesions and that FGF tunes the mechanical contrast between homophilic and heterophilic adhesions.

E-cad force transduction requires EGFR and EGF (Muhamed et al., 2016; Sehgal et al., 2018; Sullivan et al., 2022). Measurements with A431-D^E-cad^ cells tested whether E-cad-mediated traction generation similarly depended on immobilized cadherin ligand and EGFR. In low-serum medium, A431-D^E-cad^ cells exerted significantly higher RMS traction on E-cad than on N-cad-coated gels (160±65 Pa and 82±14 Pa, respectively) (Fig. 4B,D; Fig. S3C). Inhibiting EGFR with Gefitinib reduced the traction forces on E-cad to the same level as on N-cad substrates (87±13 Pa) (Fig. 4D, Fig. S3A). In controls, Gefitinib did not alter A431-D^E-cad^ traction forces on collagen-coated gels (Fig. S3D). Stimulating EGFR with 100 ng ml^−1^ EGF or with 10% serum further increased the RMS traction on E-cad substrates, but not on heterophilic N-cad-coated gels (Fig. S3C). These results show that E-cad traction forces on homophilic ligands are dependent on EGFR and EGF, and EGF stimulation enhances the contrast between cell tractions on homophilic E-cad versus heterophilic N-cad gels.

To verify that cell spreading was qualitatively similar on cadherin-coated glass and on gels, we quantified the spread areas of cells on the same gels as in traction measurements. Data for both traction and cell area measurements were often determined with the same cell or with cells on the same gel. The trends were similar to those for cells on glass, but there were differences. Spreading was slightly lower on the gels, as expected because spreading typically decreases with substrate rigidity. In low serum, the spread areas of A431D^Ncad+FGFR^ cells were similar on N-cad and E-cad gels. However, 100 ng ml^−1^ FGF or 10% serum significantly increased spreading on N-cad gels, but not on E-cad (Fig. S2C). Likewise, in low serum, A431-D^Ecad^ areas were similar on E-cad and N-cad gels. However, adding 100 ng ml^−1^ EGF or 10% serum increased spreading on homophilic E-cad, but not on N-cad gels (Fig. S2D). The results in Fig. S2C,D show that cells preferentially spread on the softer homophilic cadherin substrates, but the manifestation of ligand-selective spreading on gels requires higher growth factor concentrations than on glass.

### Differential α-catenin unfurling on homophilic versus heterophilic cadherin substrates

**Fig. 5.**
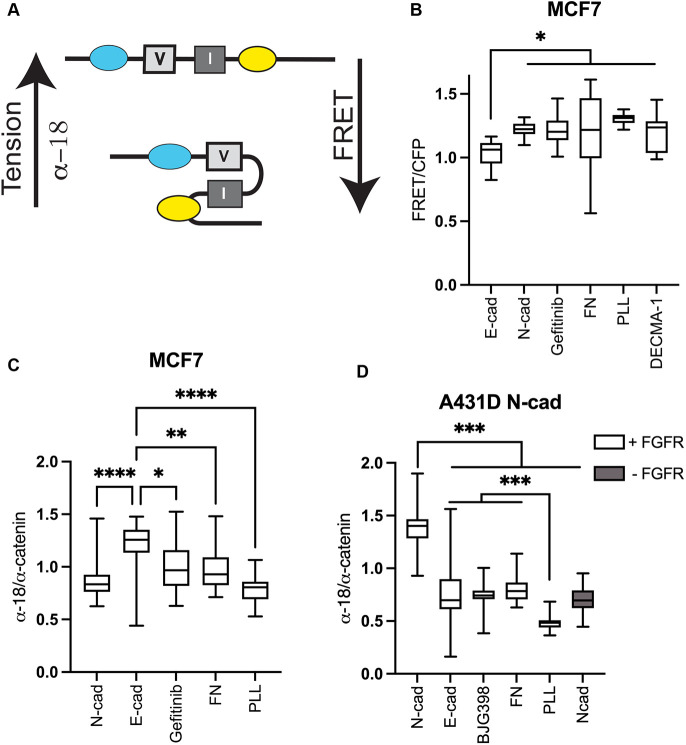
**α-catenin activation depends on cadherin ligand and RTK activity.** (A) Schematic of force-dependent conformation changes in α-catenin in the FRET-based conformation sensor (CFP and YFP are indicated by cyan and yellow, respectively). At low tension, the vinculin-binding site (V) is encrypted by an inhibitory interface (I), and increased tension exposes the vinculin site. This change decreases the FRET signal, but it exposes the α-18 epitope and increases the α-18/α-catenin ratio. (B) FRET/CFP intensity measured with MCF-7 cells on glass coated with E-cad, N-cad, fibronectin (FN), poly(L-lysine) (PLL) or DECMA-1. Data are also shown for Gefitinib-treated MCF-7 cells on E-cad. Number of cells per condition: *n*_E-cad_=*n*_N-cad_=22, *n*_FN_=14, *n*_PLL_=15, *n*_DECMA-1_=11; *N*_exp_=3. (C) α-18/α-catenin ratios determined from immunofluorescence images of MCF-7 cells on glass coated with E-cad, N-cad, FN, PLL, or DECMA-1. Data are also shown for Gefitinib-treated MCF-7 cells on E-cad. Number of cells per condition: *n*_E-cad_=40 *n*_N-cad_=40, *n*_FN_=43, *n*_PLL_=16, *n*_Gefitnib_=39; *N*_exp_=3. (D) α-18/α-catenin ratios in A431-D^N-cad±FGFR^ cells on glass coated with E-cad, N-cad, fibronectin (FN), or poly(L-lysine) (PLL). Data are shown for A431D^N-cad±FGFR^ cells on N-cad that were treated with BGJ398. The gray box shows data obtained with A431-D^N-cad^ cells (−FGFR expression) on N-cad coated glass. Number of cells per condition: *n*_N-cad_=38, *n*_E-cad_=44, *n*_FN_=38, *n*_PLL_=45, *n*_BGJ398_=38, *n*_N-cad+FGFR_=49; *N*_exp_=2. For box plots, the box represents the 25–75th percentiles, and the median is indicated. The whiskers show the complete range. *P<0.05; **P<0.005; ***P<0.0005; ****P<0.0001 (unpaired two-tailed *t*-test for 1:1 comparisons; one-way ANOVA for multiple comparisons).

The force-activated conformational change of α-catenin (Fig. 5A) is a signature of cadherin force transduction, and its activation state depends on tension across cadherin bonds (Kim et al., 2015; Yao et al., 2014; Yonemura et al., 2010). Studies next tested whether the cadherin-mediated traction forces (Fig. 4) produced corresponding differences in α-catenin activation. Initial measurements used MCF-7 cells transiently transfected with a FRET-based, α-catenin conformation sensor (Kim et al., 2015). In this sensor, α-catenin unfurling separates the fluorophores and decreases the FRET/CFP ratio (Fig. 5A). This sensor binds cadherins and actin reports conformation changes in response to junctional tension (Kim et al., 2015). Fig. 5B compares FRET/CFP ratios measured over the entire MCF-7 cell on protein-A-coated glass with immobilized E-cad-Fc or N-cad-Fc. Based on modified ELISA assays (Qin et al., 2020), the densities of immobilized E-cad-Fc and N-cad-Fc were similar at 3.32×10^−11^ and 3.31×10^−11^ mol cm^−2^, respectively. The normalized FRET/CFP ratios were 1.0±0.1 and 1.22±0.05 (mean±s.d.) on E-cad- and N-cad-coated glass, respectively (Fig. 5B). The higher FRET/CFP in MCF-7 cells on N-cad shows that there was less α-catenin unfurling than on homophilic E-cad surfaces, consistent with trends in traction force data. Gefitinib treatment increased the normalized FRET/CFP ratio to 1.20±0.12, which was similar to that for cells on N-cad substrates (Fig. 5B). In controls, normalized FRET/CFP ratios in MCF-7 cells on FN- or on PLL-coated substrates (Fig. 5B) were 1.20±0.29 and 1.30±0.04, respectively. In controls with cells bound to DECMA-1, the FRET/CFP ratio was 1.19±0.14 (Fig. 5B), which is like cells on N-cad- or on FN-coated glass. Thus, relative to MCF-7 cells on E-cad substrate, FRET was higher (less α-catenin activation) under all other conditions investigated. Tension alone is sufficient to unfurl α-catenin (Kim et al., 2015; Yao et al., 2014), so the differences in FRET ratios are attributed to ligand-selective differences in traction (see Fig. 4).

FRET measurements were confirmed by immunostaining with the α-18 antibody, which recognizes an epitope near the vinculin-binding site of α-catenin that is unmasked under tension (Nagafuchi et al., 1991; Yonemura et al., 2010). Higher ratios of α-18/α-catenin reflect greater α-catenin activation (Fig. 5A). Representative immunofluorescence (IF) images of MCF-7 cells are in Fig. S4. Owing to the different quantum efficiencies of the fluorophores on the secondary antibodies used, the ratios do not reflect the stoichiometry of unfurled to folded α-catenin.

The α-18/α-catenin ratios at the basal planes of MCF-7 cells were higher in cells on E-cad (1.14±0.32) than on N-cad substrates (0.87±0.17) (Fig. 5C), consistent with the FRET data (Fig. 5B). On control FN- and PLL-coated surfaces, α-18/α-catenin ratios were 0.97±0.19 and 0.77±0.12, respectively (Fig. 5C). Gefitinib treatment reduced α-18/α-catenin levels in cells on E-cad substrates to 0.99±0.03, which is statistically similar to that of cells on FN (Fig. 5C). In A431-D^N-cad+FGFR^ cells, α-catenin activation was similarly higher on homophilic N-cad (1.37±0.18) versus heterophilic E-cad substrates (0.77±0.25) (Fig. 5D). The FRET-based α-catenin sensor was not used with A431-D^N-cad+FGFR^ cells because the expressed GFP–FGFR1 interferes with the FRET measurements.

As in traction measurements, FGFR1 co-expression with N-cad enhanced the difference in α-catenin activation on N-cad versus E-cad substrates (Fig. 5D). With A431-D^N-cad^ cells (without FGFR) on N-cad-coated glass, the α-18/α-catenin ratio was 0.70±0.11, which is statistically similar to BGJ398 treated A431-D^N-cad^ cells on N-cad (0.75±0.10, Fig. 5D). In controls with A431-D^N-cad+FGFR^ cells on FN or PLL, the ratios were, respectively, 0.79±0.11 and 0.47±0.05 (Fig. 5D). Comparisons of results with A431-D^N-cad+FGFR^ versus A431-D^N-cad^ cells show that FGFR1 co-expression enhances differences in N-cad-mediated α-catenin activation on N-cad versus E-cad substrates, consistent with traction force data.

There are potential pitfalls of whole-cell IF imaging, because α-catenin could be in Golgi or bound to actin, as well as at junctions. Such distributions should be similar in N- cadherin- and E-cad-expressing A431-D cells. Thus, measured global differences in α-18/α-catenin ratios determined with different cadherin substrates are expected to reflect differences in tension at substrate adhesions.

### RhoA activation in cells on cadherin substrates depends on cadherin ligation and RTK activity

Cadherin adhesion activates RhoA GTPases (Braga et al., 1999), which regulate traction forces and junctional tension (Acharya et al., 2018; Kong et al., 2022; Ratheesh et al., 2012). To determine whether adhesion-dependent increases in RhoA activity correlate with cell tractions and α-catenin activation, both a FRET-based RhoA sensor (Yoshizaki et al., 2003) and a G-LISA kit were used to quantify ligand-dependent RhoA activation on 40 kPa PAA gels, as used in traction force measurements. In prior studies, RhoA-GTP levels peaked 90 min after seeding cells on cadherin surfaces (Tabdili et al., 2012a). In other studies, GTPase activity continued to increase for at least 60 min after seeding cells on cadherin modified substrates (Noren et al., 2001). RhoA activity was thus measured 90 min after cell seeding.

**Fig. 6.**
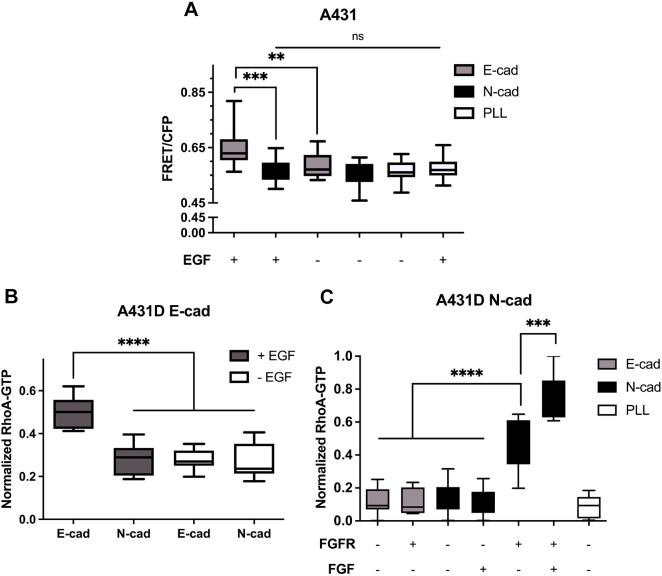
**RhoA activation depends on cadherin ligation and growth factor activation.** (A) FRET/CFP ratio of the RhoA sensor in A431 cells on 40 kPa gels modified with E-cad (gray), N-cad (black) or PLL (white), under low serum (0.5%) conditions. The *x*-axis labels indicate added 100 ng ml^−1^ EGF (+/−). Measurements were done with cells on E-cad (gray), N-cad (black) or PLL control (white) substrates. Number of cells per condition: *n*_E-cad_=34, *n*_N-cad_=31, *n*_E-cad+EGF_=33, *n*_N-cad+EGF_=33, *n*_PLL_=34, *n*_PLL+EGF_=34; *N*_exp_=3. (B) Rho-GTP/total Rho ratios measured with A431D^E-cad^ cells on 40 kPa gels coated with E-cad-Fc or N-cad-Fc in 0.5% serum, with (gray) or without (white) 100 ng ml^−1^ EGF, determined with a G-LISA assay. Number of gels per condition: *n*_E-cad_=8, *n*_N-cad_=*n*_E-cad+EGF_=*n*_N-cad+EGF_=9; Replicates *N*_exp_=3. (C) RhoA-GTP/total RhoA determined with A431D^N-cad^ cells on 40 kPa gels coated with E-cad (gray), N-cad (black) or PLL (white) in 0.5% serum, determined by G-LISA assay. The first line under the x-axis indicates FGFR co-expression (+/−). The second line indicates added 100 ng ml^−1^ FGF (+/−). Number of gels per condition: *n*=9. Replicates *N*_exp_=3. For box plots, the box represents the 25–75th percentiles, and the median is indicated. The whiskers show the complete range. **P<0.005; ***P<0.0005; ****P<0.0001; ns, not significant (unpaired two-tailed *t*-test for 1:1 comparisons; one-way ANOVA for multiple comparisons).

E-cad–GFP in A431-D^E-cad^ cells interferes with FRET measurements, so FRET studies of RhoA activation by E-cad used A431 cells, which express endogenous E-cad and overexpress EGFR. A431 cells were transiently transfected with the RhoA sensor (Yoshizaki et al., 2003) (Fig. S5A). At 90 min after seeding, there was significant RhoA activation in cells on E-cad-coated gels. In low-serum medium, the FRET/CFP ratios in A431 cells on either E-cad- or N-cad-coated 40 kPa gels were statistically similar at, respectively, 0.58±0.04 and 0.56±0.04 (mean±s.d.; Fig. 6A). However, adding 100 ng ml^−1^ EGF increased the FRET/CFP ratio in cells on E-cad (0.64±0.06) but not on N-cad gels (0.57±0.04) (Fig. 6A). EGFR activation thus enhanced ligand-dependent differences in RhoA activation. EGF concentrations had no effect on FRET/CFP ratios in cells on control PLL-coated gels (Fig. 6A). These results demonstrate RhoA activation at E-cad adhesions is ligand dependent (Fig. 6A).

A G-LISA kit was used for RhoA assays with cells that expressed fluorescently labeled cadherin or FGFR. With A431 cells, we obtained similar results using the RhoA-FRET and G-LISA kit (Fig. 6A, Fig. S5B). G-LISA measurements with A431-D^E-cad^ cells on E-cad- versus N-cad-coated gels also agreed with FRET data obtained with A431 cells (Fig. 6B; Fig. S5B). Under low-serum conditions, RhoA-GTP/total RhoA levels in A431-D^E-cad^ cells were statistically similar on both E-cad- and N-cad-coated gels (Fig. 6B), but 100 ng ml^−1^ EGF increased RhoA activation more than twofold in cells on E-cad gels (Fig. 6B). EGF had no effect on RhoA in A431-D^E-cad^ cells on N-cad (Fig. 6B). EGF also had no effect on A431 cells on either control PLL- or N-cad-coated gels (Fig. S5B).

N-cad-mediated RhoA activation also depends on the immobilized cadherin ligand, FGFR and FGF. RhoA-GTP levels in A431-D^N-cad^ cells (which do not express FGFR1) seeded on E-cad or N-cad-coated gels were statistically similar (Fig. 6C), and FGF had no effect in either case. The latter RhoA-GTP levels were similar to those for control cells on PLL (Fig. 6C). Compared with A431-D^N-cad^ cells, FGFR expression increased active RhoA in A431-D^N-cad+FGFR^ cells on N-cad, in low-serum medium without added FGF (Fig. 6C). This result agrees with reports that N-cad binds and activates FGFR at the membrane (Suyama et al., 2002; Williams et al., 2001). By contrast, FGFR expression had no effect on RhoA activation when A431-D^N-cad+FGFR^ cells were seeded on E-cad (Fig. 6C) or on PLL (Fig. S5C). Adding FGF further increased RhoA-GTP in A431-D^N-cad+FGFR^ cells on N-cad substrates (Fig. 6C). The FGFR inhibitor BGJ398 reduced RhoA-GTP to control levels, in the presence and absence of FGF (Fig. S5C). These results support the hypothesis that FGFR1 cooperates with N-cad to activate RhoA-GTP in cells on homophilic N-cad substrates, but FGFR expression and FGF have no effect on cells that were seeded on heterophilic E-cad or control PLL surfaces. These results suggest that N-cad cooperates with FGFR to switch on RhoA activation at homophilic, but not heterophilic cadherin adhesions.

### Cadherin ligation cooperates with RTKs to selectively regulate force transduction signaling

We further investigated other ligand-dependent signaling. Tension on E-cad receptors on epithelial cells activates EGFR (Muhamed et al., 2016; Sehgal et al., 2018; Sullivan et al., 2022). To test whether the mechanical activation of EGFR depends on cadherin ligation, western blotting was used to assess phosphorylation at tyrosine 845 (pY845) after mechanically perturbing E-cad receptors under similar conditions as in the MTC measurements (Sullivan et al., 2022). Fig. S6A shows a representative western blot, and the Y845/EGFR ratios after perturbing cells with N-cad or E-cad beads are in Fig. S6B. The basal level of pY845/EGFR in E-cad bead-laden cells appears to be lower than in cells with N-cad beads, but the difference is not statistically significant. By contrast, tugging on the E-cad beads activates EGFR phosphorylation, but tugging on N-cad beads does not. These results further support the hypothesis that only homophilic E-cad adhesions can mechanically activate EGFR.

**Fig. 7.**
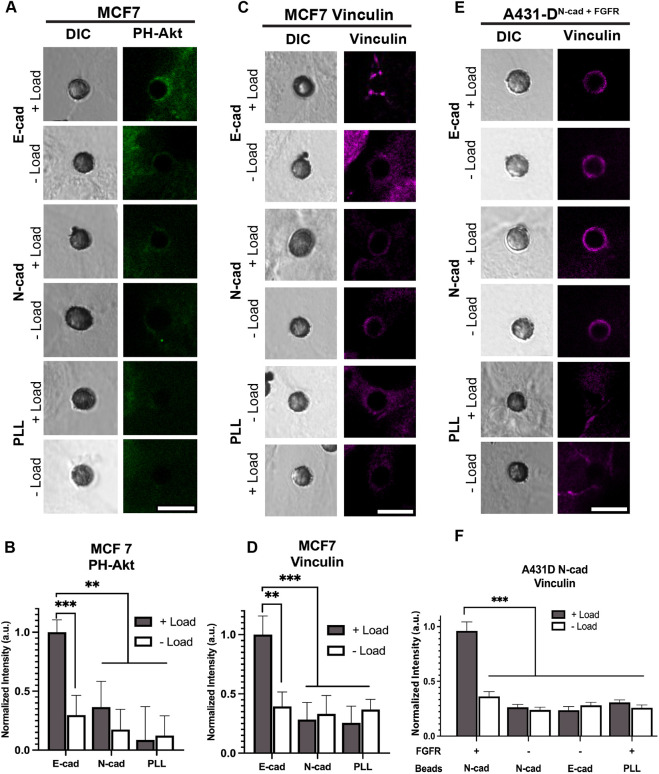
**Mechanical activation of PI3K and vinculin recruitment to cadherin-modified beads on MCF-7 and A431D^Ncad+FGFR^ cells requires homophilic ligation.** (A) DIC images of beads on MCF-7 cells and confocal immunofluorescence images of PH-Akt–GFP in ROIs around the bead-cell junctions, with (+Load) and without (−Load) bead twisting. Data are shown for measurements with E-cad, N-cad or PLL coated beads. Scale bars: 8 μm. (B) Graph of the fluorescence intensities from analyses of beads as in A, normalized by the intensity at perturbed (+Load) E-cad beads. The *x*-axis indicates the bead coating. Beads analyzed per condition: *n*_E-cad_=45, *n*_n-cad_=50, *n*_PLL_=50; replicates *N*_exp_=2. (C) DIC images of beads on MCF-7 cells and confocal immunofluorescence images of vinculin at beads coated with E-cad, N-cad or PLL, with (+Load) or without (−Load) bead twisting. (D) Quantified fluorescence intensities from analyses of beads in part C, normalized by the intensity at perturbed (+Load) E-cad beads. The *x*-axis indicates the bead coating. *n*_beads_=45 for each condition; replicates *N*_exp_=2. (E) DIC images of beads on A431D^Ncad+FGFR^ cells and confocal IF images of vinculin at beads coated with E-cad, N-cad, or PLL, with (+Load) or without (−Load) bead twisting. (F) Graph of vinculin fluorescence intensities around beads on A431-D^Ncad^ cells, normalized by the intensity at perturbed (+Load) N-cad beads on A431-D^Ncad^ cells expressing FGFR (A431D^Ncad+FGFR^). First line under the *x*-axis indicates FGFR expression (+/−) and the second line indicates the bead coating. *n*_E-cad_=50, *n*_n-cad_=49, *n*_PLL_=50, *n*_N-cadFGFRload_=75, *n*_N-cadFGFRnoload_=30; replicates *N*_exp_=2. Data in B, D and F are the mean±s.e.m. **P<0.005; ***P<0.0005 (unpaired two-tailed *t*-test for 1:1 comparisons; one-way ANOVA for multiple comparisons).

E-cad force transduction activates phosphoinositide-3-kinase (PI3K) downstream from EGFR (Sehgal et al., 2018), so studies next compared PI3K activation by tugging on cells with E-cad- versus N-cad-coated beads. PH-Akt–GFP is a location reporter for active PI3K (Kwon et al., 2007). Fluorescence measurements of PI3K activation at E-cad beads was undertaken with MCF-7 cells, because A431-D^E-cad^ cells express GFP-tagged E-cad. Fig. 7A shows representative DIC and fluorescence images of regions of interest (ROIs) around E-cad- or N-cad beads on MCF-7 cells, with and without applied force. Changes in PH-Akt–GFP intensities were scaled to the GFP intensity at perturbed E-cad-beads, and results were averaged over ROIs at >45 beads per condition. Tugging with E-cad beads increased PH-Akt–GFP, relative to no-load and PLL-coated bead controls (Fig. 7A,B). However, at N-cad beads, the PH-Akt–GFP levels were slightly higher than PLL controls, but lower than at E-cad beads (Fig. 7B).

Actin and vinculin recruitment to stressed E-cad adhesions is downstream from RTK signaling (Kong et al., 2022; Muhamed et al., 2016; Sehgal et al., 2018). If heterophilic cadherin adhesions fail to activate signaling and cell stiffening, we predicted they would not activate vinculin or actin recruitment. Immunofluorescence imaging quantified force-dependent actin and vinculin recruitment at homophilic versus heterophilic adhesions. The quantitative results were scaled to the average actin or vinculin at beads after twisting, tugging and compared with no-load controls. With MCF-7 cells, increased force resulted in actin and vinculin accumulation at E-cad beads, in contrast with the negligible changes at N-cad or control PLL beads (Fig. 7C,D; Fig. S7A,B).

Similar measurements were done with N-cad-expressing cells. Representative IF images of vinculin and actin at beads on A431-D^N-cad+FGFR^ cells showed that applied force increased actin and vinculin at N-cad beads relative to that for no-load controls (Fig. 7E,F; Fig. S7C,D). However, with A431-D^N-cad^ cells, which do not express FGFR, N-cad beads did not trigger vinculin or actin recruitment (Fig. 7F), consistent with the absence of cell stiffening (Fig. 2C). The N-cad surface expression was similar on both A431-D^N-cad+FGFR^ and A431-D^N-cad^ cells, so the difference is attributed to cooperation between FGFR1 and N-cad. By contrast, E-cad beads on A431-D^N-cad+FGFR^ cells failed to activate either vinculin or actin recruitment (Fig. 7E,F; Fig. S7C,D).

## DISCUSSION

**Fig. 8.**
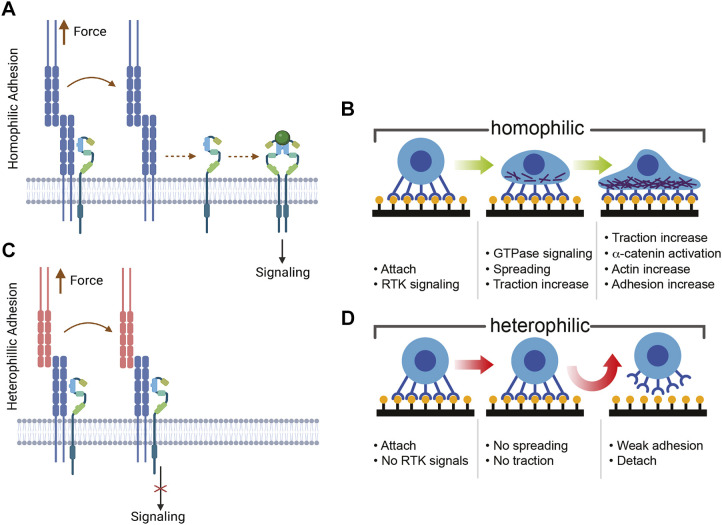
**Proposed model of cadherin mechano-selectivity and downstream effects of ligation-selective signal activation.** (A) Cadherins associate with RTKs on the cell surface. Tension on homophilic cadherin bonds releases the RTK for activation and signaling. (B) Signaling initiates cascades that increase cell contractility and actin remodeling at junctions to stabilize cadherin adhesions. (C) At heterophilic cadherin bonds, tension does not activate RTK-mediated signaling. (D) The absence of force-transduction signaling at heterophilic junctions eliminates downstream contractility and reinforcement of stressed junctions, such that cadherin adhesions are not reinforced, and cells detach more readily from heterophilic cadherin substrates. Panels A and C were created in BioRender. Leckband, D., 2025. https://BioRender.com/d57t444. Republished with permission.

Two main findings of this study are that (1) N- and E-cad cooperate with specific growth factor receptors (RTKs) to form signaling mechano-switches at intercellular junctions and (2) that force-activated RTK signaling requires homophilic cadherin ligation, independently of cadherin binding affinities. Prior studies have reported large differences in the amplitude of cell stiffening in response to tugging with homophilic ligand versus heterophilic ligand or antibody-coated beads (Barry et al., 2014, 2015; Tabdili et al., 2012a,b). Cadherin mechano-selectivity has been documented with four different classical cadherins and at least five different cell types (Tabdili et al., 2012b). The role of α-catenin in cadherin force transduction is well-established (Ito et al., 2017), but its activation depends on force, independently of cadherin identity (Kim et al., 2015; Yao et al., 2014). Here, new findings show that the cadherin-mediated cell stiffening requires specific RTKs, and soluble growth factor amplifies the force transduction response and the contrast between heterophilic and homophilic ligands. These and prior results further support the hypothesis that homophilic cadherin ligation is the key that selectively unlocks force-activated, RTK-dependent signaling (Fig. 8A) (Barry et al., 2015; Tabdili et al., 2012a,b).

This study did not determine how cadherins activate RTKs, but one hypothesis supported by experimental evidence is that it involves a direct force-sensitive interaction (Coon et al., 2015; Leckband, 2023; Sullivan et al., 2022; Williams et al., 2001). Loss of force-transduction following EGFR knockdown (Sehgal et al., 2018) and the gain of function upon co-expression of N-cad and FGFR supports this, in part, but there is evidence that cadherins directly bind and regulate RTKs. FRET measurements have shown that E-cad forms heterotrimeric complexes with EGFR on membranes of live cells (Sullivan et al., 2022). The fourth extracellular region of N-cad, EC4 binds an auto-inhibitory ‘acid box’ on FGFR, to activate signaling and regulate intercellular adhesion (Nguyen et al., 2019; Sanchez-Heras et al., 2006; Williams et al., 2001). VE-cadherin associates with VEGFR2 and VEGFR3 at endothelial junctions to regulate flow sensing (Coon et al., 2015). Furthermore, tension disrupts both E-cad–EGFR and VE-cadherin–VEGFR2 complexes, resulting in RTK activation (Sullivan et al., 2022; Tian et al., 2016; Kong et al., 2022). Less is known about the effect of N-cad tension on FGFR. Other junctional proteins could be involved, but the above evidence and the disruption of force transduction by RTK inhibitors or growth factor depletion support the view that RTKs are key components of cadherin force transduction machinery.

Direct hetero-receptor contacts raise the possibility that *trans* cadherin binding could allosterically regulate the cadherin–RTK interface. Growth factor receptors are allosteric proteins (Lemmon et al., 2014). In cadherin complexes, the cytosolic protein p120 catenin allosterically regulates E-cad *trans* binding, as do antibodies targeting epitopes distal from the cadherin binding interface (Ozawa et al., 1990; Petrova et al., 2012; Shashikanth et al., 2015). Similarly, activating VE-cadherin force transduction with antibody-coated beads depended on the targeted epitope (Barry et al., 2015). Future studies will test this hypothesis.

Importantly, specific ligand recognition determines cadherin-mediated signaling amplitudes that do not scale with the binding affinities. Cadherin mechano-selectivity thus supersedes differences in adhesion energies. The contrasts in RTK-dependent signaling amplitudes thus generates correspondingly large differences in cell spreading, cytoskeletal remodeling, contractility and traction generation on heterophilic versus homophilic cadherin ligand (Fig. 8B). The lack of actin reinforcement on heterophilic cadherin substrates also correlates with significantly lower cell attachment densities (Fig. 8B). Affinity differences do not explain these differences, but cadherin mechano-selectivity does.

Cadherin mechano-selectivity would also account for the lack of adhesion between cells expressing different cadherin subtypes in micropipette measurements (Chu et al., 2004). This behavior might also impact tissue surface tension, which influences cell segregation in multicellular aggregates (Méhes et al., 2023). Additionally, this present study focused mainly on adhesion-related cell functions, but cadherins and RTKs also cooperate to regulate other processes such as contact inhibition of proliferation (Gumbiner and Kim, 2014; Leckband, 2023). The findings could have broader implications for other cell functions.

The tendency for cells expressing different cadherins to segregate in multicellular aggregates is frequently attributed to stronger homotypic cadherin bonds, in contradiction with biophysical measurements (Katsamba et al., 2009; Niessen and Gumbiner, 2002; Prakasam et al., 2006). Cortical tension is also a major organizing factor (Campas et al., 2023). Our findings might reconcile these conflicting observations, by linking mechano-selective cadherin signaling to ligand-dependent differences in cell mechanics and preferential reinforcement and stabilization of homophilic cell adhesion (Fig. 8B). The results do not negate the potential physiological significance of heterophilic contacts (Fontenete et al., 2017; Labernadie et al., 2017), but they highlight signaling and mechanical differences that could influence multicellular behavior.

In addition to EGFR and FGFR, E- and N-cad might also interact with different RTKs in other contexts (Leckband, 2023). E-cad might interact with other ErbB family members, and it associates with Met when overexpressed (Qian et al., 2004). E-cad additionally interacts with insulin-like growth factor receptor (Qian et al., 2004), and aids embryo compaction during murine blastocyst formation (Bedzhov et al., 2012). Finally, N-cad interacts with platelet-derived growth factor receptor (Theisen et al., 2007). Different cadherin–RTK interactions could regulate context-dependent mechanical functions. For example, VE-cadherin cooperates with VEGFR2 and VEGFR3, to control endothelial cell alignment in different flow environments (Coon et al., 2015). Cooperation with other proteins, such as nectins (Takeichi, 2023), raises the possibility that cadherins could also mechanically regulate other proteins and their associated functions.

## MATERIALS AND METHODS

### Cell lines, protein production and inhibitors

Michigan Cancer Foundation-7 (MCF-7) human epithelial breast carcinoma and immortalized C2C12 mouse myoblast cells were from ATCC. The A-431 and A431-D cells were a gift from Prof. Keith Johnson (University of Nebraska, Lincoln, USA) and were used as received (Lewis et al., 1997). A plasmid containing the full-length human E-cad fused to GFP at the C-terminus (deposited by Dr Jennifer Stow, Addgene plasmid #28009) was used to generate stable A431-D cells expressing E-cad–GFP (A431-D^E-cad^) (Muhamed et al., 2016). A431-D cells were stably transfected with the pcDNA3.1 plasmid containing the full-length wild-type human N-cad insert (gift from Huabei Guo, Georgia State University, Athens, GA, USA) to produce A431-D^N-cad^ cells, as described for generating A431-D^E-cad^ cells. A431-D^E-cad^ cells were selected for populations with similar median (E-cad–GFP) fluorescence intensities and comparable cadherin expression, using fluorescence activated cell sorting (FACS) (FACS ARIA II sorter, Edward R. Madigan Laboratory, Illinois). Cell surface expression was determined by quantitative flow cytometry, as described previously (Chien et al., 2008). Stable A431-D^N-cad+FGFR^ cells were made by transfecting stable A431-D^N-cad^ cells with a pCMV3 plasmid encoding the GFP-tagged FGFR1 (Sino Biological, Inc, Wayne, PA; #HG10615-ACG), followed by selection for similar median FGFR–GFP fluorescence intensities with FACS. The transfected cell lines were maintained in 200 μg ml^−1^ G418 (Teknova) and 200 μg ml^−1^ Hygromycin B (Thermo Fisher Scientific).

The A431-D^E-cad^ and A431-D^N-cad^ cells were cultured in Dulbecco's modified Eagle's medium (DMEM with 4.5 g/l glucose; Corning 50003) supplemented with 10% (v/v) fetal bovine serum (FBS, Avantor no. 76419-584), 1 mM sodium pyruvate (DMEM Corning 50003), 1% (v/v) penicillin-streptomycin (Corning Cell Grow, Manassas, VA, USA) and 200 μg ml^−1^ G418 (GoldBio CAS108321-42-2). A431-D^N-cad+FGFR^ were cultured in DMEM (4.5 g/l glucose) supplemented with 10% (v/v) FBS, 1 mM sodium pyruvate, 1% (v/v) penicillin-streptomycin, 200 μg ml^−1^ G418 and 200 µg ml^−1^ Hygromycin B (Goldbio CAS 31282-04-9). A431-D^N-cad^, A431-D^N-cad+FGFR^ and A431-D^E-cad^ were verified via flow cytometry and tested negative for myoplasm contamination. The MCF7 line was also verified with flow cytometry. The C2C12 cell line was verified via differentiation assays. Soluble, recombinant E-cad and N-cad extracellular regions fused to C-terminal Fc domains of human IgG were produced as described (Prakasam et al., 2006). The noncompetitive EGFR-specific inhibitor Gefitinib (IRESSA) and FGFR1-specific inhibitor BJ NVP-BGJ398 (S2183) were from SelleckChem (Houston, TX, USA). Gefitinib was used at a concentration of 15 μM and BGJ398 at 2 μM. The inhibitors were added 2 h prior to the measurements.

### Magnetic twisting cytometry

Ferromagnetic beads (5 µm diameter, Spherotech) were modified with Fc-tagged extracellular domains of E-cad or N-cad and incubated with confluent cell monolayers cultured on FN-coated glass-bottomed dishes. We used glass substrates because they ensure the best bead imaging quality. After magnetizing the beads with a brief 20,000 Gauss pulse parallel to the cell plane, an orthogonal twisting torque was applied for 120 or 240 s using an oscillating field of 60 Gauss at a frequency of 0.33 Hz. The twisting torque (*T*) on the beads generates a shear stress (force/area) of ∼7 Pa on the membrane-bound cadherin receptors. To record force-dependent changes in cell stiffness, the bead displacements (*D*) were imaged with an inverted microscope (Zeiss) equipped with a 20×0.6 NA objective. The relative changes in the moduli of the bead–cell junction were determined from changes in bead displacement amplitudes during twisting. In all cases, the bead coatings were optimized to bind cells and undergo displacements sufficient to quantify the modulus (le Duc et al., 2010). For control experiments, beads were coated with poly-L-Lysine (PLL, Sigma St Louis, MO, USA) or anti-E-cad antibody, DECMA-1 (Sigma). For each condition, at least 150 beads (one bead per cell) were analyzed for statistical significance. The data follow a log-normal distribution, from which we obtain the mean and standard deviation. The reported cell stiffness values, relative to the initial stiffness, are the mean±s.d. Changes are assessed using a one-tailed unpaired *t*-test, with *P*<0.05 indicating statistical significance.

### Confocal IF imaging

In MTC studies, proteins were visualized at bead–cell contacts by IF imaging (Barry et al., 2014; le Duc et al., 2010; Muhamed et al., 2016). Immediately after bead twisting, cells were fixed with 4% (w/v) paraformaldehyde for 15 min at room temperature. Control cells were not subjected to bead twisting, but all conditions were otherwise identical. Cells were permeabilized with 0.1% Triton X-100 for 5 min, blocked in 1% (w/v) BSA for 20 min. PI3K was visualized with PH-Akt–GFP (pcDNA3-AKT-PH-GFP was Addgene plasmid #18836, deposited by Craig Montell). Vinculin was visualized by immunostaining overnight with primary rabbit anti-vinculin antibody (1:200, CST Beverly, MA), followed by rinsing, and treatment with secondary antibody goat anti-rabbit-IgG conjugated to Texas red (1:200, Invitrogen, Carlsbad, CA, USA). Actin was stained with phalloidin–Alexa Fluor 647 (1:250, Life Technology, Carlsbad, CA, USA) in 1% (w/v) BSA for 1 h. The DIC and fluorescence images were acquired with laser-scanning confocal microscope (LSM700, Zeiss, Carl Woese Institute for Genomic Biology, UIUC). Protein recruitment in regions of interest (ROIs) was quantified with ImageJ by drawing a ring around the bead 1 µm from the bead edge. Background fluorescence for all images was determined in a region outside of the cell, and the background-subtracted fluorescence intensities were normalized by the intensity measured at force-loaded homophilic adhesions in the presence of serum or growth factor.

In measurements of α-catenin activation in cells on cadherin substrates, after fixation, α-catenin was labeled with the conformation-specific, anti-α-catenin α-18 antibody (1:150, a gift from Akira Nagafuchi, Nara Medical University, Nara, Japan; Nagafuchi et al., 1991; Yonemura et al., 2010) followed by FITC-labeled goat anti-rat-IgG antibody (1:200, Invitrogen). The α-18 intensity was normalized by total α-catenin, which was determined with primary rabbit anti-α-catenin antibody (1:200, Sigma), followed by labeling with secondary Texas Red-labeled goat anti-rabbit-IgG antibody (1:200, Invitrogen).

### Western blotting

Western blotting was used to assess FGFR1 expression in A431-D^N-cad+FGFR^ and in A431-D^N-cad^ cells. Cell lysates were separated by SDS-PAGE and transferred to membranes as described above. After blocking, membranes were incubated at 4°C overnight with rabbit anti-FGFR1 antibody (1:1000, Cell Signaling Technology, Beverly, MA, USA). Actin loading controls were stained with mouse anti-actin antibody (1:2000, BD Transduction Franklin Lakes, NJ, USA) in Tris-buffered saline (TBS) containing 10% (w/v) BSA. After incubation, the membrane was washed thrice with TBST, incubated with horseradish-peroxidase-conjugated anti-rabbit IgG (1:5000, Sigma St. Louis, MO) or anti-mouse IgG (1:3000, Promega) for 1 h. Blots were then visualized with an enhanced chemi-luminescence system (Amersham, Arlington Heights, IL, USA). Images can be found in Fig. S8.

To quantify EGFR activation in MTC experiments, cells were immediately lysed with Laemmli sample buffer (BioRad, #1610737EDU) supplemented with 5% (v/v) 2-mercaptoethanol and phosphatase inhibitor cocktails (Sigma, P0001-1ML). Cell lysates were then subjected to a 10 min boiling at 95°C, followed by centrifugation at 15,000 ***g*** for 10 min to remove cellular debris and beads. The resulting samples were analyzed by SDS-PAGE using a 4–20% gradient gel (BioRad, 4561096). A semi-dry western blot transfer was conducted using PVDF membranes at 10 V for 45 min. Subsequently the membrane was incubated overnight at 4°C with anti-pY845 EGFR (1:1000, Cell Signaling Technology, #2231) in 5% (w/v) BSA in TBST (10 mM Tris-HCl pH 7.4, 150 mM NaCl and 0.05% Tween-20). Mouse anti-actin (1:2000, BD Transduction, Ab-5) in with 5% (w/v) BSA was used for the actin loading control. After incubation, the membrane was washed three times with TBST and incubated with horseradish-peroxidase-conjugated anti-rabbit IgG (1:5000, Invitrogen, #31460) or anti-mouse IgG (1:5000, Invitrogen, #31430) for 90 min at room temperature. The membrane was washed three times with TBST and then visualized with an enhanced chemiluminescence system (Azure Biosystems, AZI400-01). Following imaging, the blot was stripped with stripping buffer (Thermal Fisher Scientific, 21059) for 45 min at room temperature and then washed twice with TBST for 5 min before re-probing overnight at 4°C with anti-EGFR (1:1000 dilution, Cell Signaling Technology, D38B1). After the overnight incubation, the membrane was washed three times with TBST and incubated with HRP-conjugated anti-rabbit-IgG (1:5000, Invitrogen, #31460) for 90 min, followed by washing three times with TBST and imaging. E-cad re-probing followed the above procedure with anti-E-cad antibody (1:2000, BD Bioscience, 610181). The gray scale intensity of E-cad bands, EGFR bands and pY845 bands on the blot membrane were quantified with the Gels plugin in ImageJ. Unprocessed blot images can be found in Fig. S8.

### Single-cell α-catenin activation measurements

α-catenin activation at cadherin adhesions was quantified using an α-catenin-based FRET sensor (Kim et al., 2015) and a conformation sensitive anti-α-catenin antibody, α-18, which recognizes an α-catenin epitope that is exposed under tension (Nagafuchi et al., 1994). In FRET measurements, to quantify in α-catenin activation in cells adhering to different substrates, MCF-7 cells were transiently transfected with the FRET-based α-catenin sensor using Lipofectamine 2000 (Invitrogen; Kim et al., 2015). MCF-7 cells express endogenous E-cad and normal EGFR levels. In FRET measurements, cells were seeded in medium containing 0.5% serum and 16G3 antibody (gift of Kenneth Yamada, NIH/NIDCR, Bethesda, MD, USA), at a density of 10^5^ cells ml^−1^. Glass slides were used to ensure good signal-to-noise ratios in FRET measurements. The slides were coated with physisorbed protein A at 0.02 mg ml^−1^ (Sigma), followed by rinsing and subsequent incubation with Fc-tagged E- or N-cad (0.02 mg ml^−1^). The proteins were adsorbed at 37°C for 1 h at each step, followed by three washes with phosphate-buffered saline (PBS). For controls, the slides were coated with physisorbed FN (EMD Millipore Corp) or PLL (Sigma) at 0.01 mg ml^−1^. In an additional control, DECMA-1 (0.01 mg ml^−1^; Sigma) was immobilized on protein A. The FRET/ECFP ratio of the sensor was determined from fluorescence images obtained with a Zeiss Axiovert 200 inverted microscope (Beckmann Institute, University of Illinois). ROIs (whole cells) were analyzed with MetaFluor 6.2 imaging software to assess relative differences in the α-catenin conformation at cell–substrate adhesions.

In measurements of α-catenin activation in cells adhered to cadherin substrates, after fixation, the α-catenin was labeled with the conformation-specific, anti-α-catenin α-18 antibody (1:150, gift from Akira Nagafuchi; Nagafuchi et al., 1994), followed by FITC-labeled goat anti-rat-IgG antibody (1:200, Invitrogen). The α-18 staining was normalized by total α-catenin. Total α-catenin was determined with primary rabbit anti-α-catenin antibody (1:200, Sigma), followed by labeling with secondary Texas red-labeled goat anti-rabbit-IgG antibody (1:200, Invitrogen).

### Traction force microscopy

Traction force measurements (Tolic-Norrelykke et al., 2002) were conducted with PAA hydrogels with Young's moduli of 40 kPa (Tse and Engler, 2010), embedded with 0.2 µm diameter fluorescent microspheres (FluoroSpheres, Thermo Fisher Scientific, #F8810). Protein A (Sigma) was immobilized, by incubating 0.1 mg ml^−1^ solutions with Sulfo-SANPAH-activated gels for 30 min at 37°C. Protein A immobilization was followed by rinsing with PBS. Cadherin was then immobilized and oriented on the substrate, by incubation with either Fc-tagged E-cad extracellular domains (0.1 mg ml^−1^) or Fc-tagged N-cad extracellular domains (0.1 mg ml^−1^) in immobilization buffer (100 mM HEPES, 100 mM NaCl, and 5 mM CaCl_2_ at pH 8) at 37°C for 3 h, followed by rinsing with immobilization buffer. Samples were then blocked with 15 (w/v) BSA in immobilization buffer for 30 min at 37°C. In other studies, protein A-modified gels were incubated with DECMA-1 (0.1 mg ml^−1^) in PBS. After protein immobilization, the substrates were rinsed twice with 1× PBS and sterilized by irradiation at 365 nm for at least 15 min before seeding cells.

The A431-D^N−cad^ and A431-D^N-cad+FGFR^ cells were harvested by treating confluent cell monolayers with 3.5 mM EDTA in PBS containing 1% (w/v) BSA. Cells were seeded at 6×10^3^ cells/cm^2^ onto hydrogels in DMEM containing 0.5% FBS. Serum-starved cells do not attach well to cadherin-modified gels, but including 0.5% serum improves attachment stability (Muhamed et al., 2016). The 16G3 antibody was included to prevent interference by secreted FN. Cells adhered and spread on the functionalized PAA gels for 6 h at 37°C, under 5% CO_2_. The root mean square (RMS) traction force was determined from fiduciary bead displacements, relative to the traction-free bead positions measured after cell lysis with a SDS solution. Constrained traction maps and the RMS traction stress (Pa; N/m^2^) were determined from bead displacement maps (Tolic-Norrelykke et al., 2002).

To quantify the N-cad and E-cad immobilized on the PAA hydrogels (Qin et al., 2020), unbound cadherin and the subsequent two washes of the substrate with Ca^2+^-containing PBS were collected and added to a 96-well polystyrene plate. After incubation for 1 h at 37°C, the plate was washed and incubated with 2% (w/v) BSA in dPBS for 1 h at 37°C, to block nonspecific protein adsorption. The wells were next incubated with rabbit anti-N-cad polyclonal antibody (1:600, ProteinTech Rosemont, IL, USA, 30803-1-AP) and rat anti-E-cad antibody (1:200, DECMA-1, Sigma, 30803-1-AP) overnight at 4°C. After rinsing the samples with buffer, horseradish peroxidase (HRP)-conjugated anti-rabbit-IgG and anti-rat-IgG (1:10,000, Sigma-Aldrich) was added to each well and incubated at room temperature for 1 h. A 3,3′,5,5′-tetramethylbenzidine (TMB) ELISA substrate kit (Thermo Fisher Scientific) was used as a chromogenic substrate for HRP. After incubation for 20 min, the absorbance of each well was measured at 370 nm with a microplate reader (Tecan Infinite 200 PRO).

### Cell density, spreading areas and actin images

Cell densities (cells mm^−2^) were determined by counting the number of cells in multiple 600 µm^2^ randomly selected ROIs on the substrates. Measurements were undertaken 90 min after seeding cells in 0.5% serum, in the presence of FN-blocking antibody 16G3 or, in the case of actin imaging only, integrin blocking antibody AIIB2 (1:50 dilution). The hybridoma producing AIIB2 was from Johan de Rooij (Hubrecht Institute, The Netherlands). At least three separate regions on the substrate were imaged to obtain representative statistics for cell densities over the entire sample (Rahil et al., 2016). The spread areas were determined with an ImageJ ‘measure area’ tool.

To visualize actin in cells on different cadherin ligands, 13 mm glass-bottom dishes were coated overnight at 4°C with E-cad-Fc or N-cad-Fc at 50 µg ml^−1^ in Hanks’ balanced salt solution (HBSS) with 1 mM CaCl_2_. The following morning, dishes were washed with 1 mM CaCl_2_ HBSS then blocked with 1% (w/v) BSA in 1 mM CaCl_2_ HBSS for 2 h at 4°C. Cells at 50% confluence in a T25 flask were harvested with 2 mM CaCl_2_ in TrypLE (Gibco 12604039) at 37°C for 10 min, followed by gentle scraping. Cells were pelleted, washed twice with HBSS supplemented with 5 mM EDTA and 1% BSA. After washing, cells were counted and adjusted to 50,000 cells ml^−1^ with HBSS containing 5 mM EDTA 1% BSA. Per condition, 5000 cells were then added to Eppendorf tubes with medium containing 0.5% FBS supplemented with 1:50 AIIB2 antibody to block integrins. For the DECMA-1 treatment, cells were incubated with the blocking antibody (1:50 dilution) for 10 min at room temperature, prior to seeding onto substrates at 5000 cells/dish and moving to a 37°C incubator. After 2 h at 37°C, the medium was aspirated, and the cells were rinsed with PBS. The sample was fixed with 4% paraformaldehyde in PBS for 20 min at room temperature. Following fixation, the cells were rinsed three times with PBS and stained with Rhodamine–phalloidin (Invitrogen, R415) 1:250 in 0.05% (v/v) Triton X-100 PBS at room temperature for 40 min. Following staining, the cells were rinsed three times with 0.05% Triton X-100 PBS and mounted with Prolong Gold Antifade (Thermo Fisher Scientific, P36930). Actin was imaged at the basal plane using a Zeiss LSM 900 confocal microscope at the University of Illinois Urbana-Champaign Institute for Genomic Biology (IGB). For representative images of actin in spread MCF7 cells, the total images acquired for each condition are *n*_E-cad_=55, *n*_DECMA_=56, *n*_N-cad_=55. For actin images in A431D^E-cad^ cells, *n*_E-cad_=110, *n*_DECMA_=70, *n*_N-cad_=110.

### RhoA activation

#### Fluorescence-based RhoA biosensor

RhoA activation was quantified following cell attachment to different substrates, using A431 cells that were transiently transfected with a FRET-based RhoA biosensor (Yoshizaki et al., 2003). Cells were transfected using Lipofectamine 2000 according to the manufacturer's specifications (Invitrogen). To prepare cadherin-displaying substrates, 40 kPa PAA hydrogels were modified as described for traction force measurements. Control substrates were modified with PLL.

Prior to measurements, the cells were cultured for 24 h in DMEM containing 0.5% FBS (low-serum condition). Cells were detached by treatment with 3.5 mM EDTA in PBS containing 1% (w/v) BSA. Prior to seeding the cells at a density of 6×10^3^ cells cm^−2^, the gels were rinsed with 1% (w/v) BSA in dPBS (containing Ca^2+^) for 1 h at 37°C, to block nonspecific cell binding. Cells were seeded on substrates in low serum (0.5%) medium and incubated with the substrates for 90 min. Including 16G3 antibody prevented interference by secreted FN, and low seeding densities prevented intercellular adhesion. When included, EGF was added to a final concentration of 100 ng ml^−1^ when cells were seeded on gels. FRET measurements were carried out with a Zeiss Axiovert 200M at 40× magnification (Institute of Genomic Biology, University of Illinois), and images were analyzed with MetaFluor 6.2 imaging software.

#### G-LISA

The G-LISA RhoA activation assay kit (Cytoskeleton Inc., cat. BK124) was used to verify the FRET results and quantify RhoA activity in A431-D^E-cad^ cells, which express E-cad–GFP, which interferes with FRET measurements. G-LISA is an ELISA-based kit for quantifying relative differences in the ratios of RhoA-GTP/RhoA. G-LISA measurements were performed with A431-D cells stably expressing E-cad–GFP (A431-D^E-cad^), N-cad (A431-D^N-cad^), or N-cad and FGFR1 (A431-D^N-cad+FGFR^). Prior to measurements, cells were cultured under low serum (0.5%) condition as in the RhoA FRET measurements. The RhoA-GTP/RhoA ratios were measured 90 min after seeding cells on different substrates, under identical conditions as in measurements with the RhoA-FRET biosensor. Fluorescence measurements were done with an ICyte Automated Imaging Cytometer (Thor Labs, Woese Institute for Genomic Biology).

### Shear flow adhesion assay

The setup is similar to that described previously (Chappuis-Flament et al., 2001). The inner walls of 76 mm long borosilicate glass capillary tubes with 1.0 mm internal diameter (Precision Instrument, Sarasota, FL; #TW150-3) were coated overnight in a humidified chamber at 4°C with 50 µg ml^−1^ cadherin-Fc (E- or N-cad) in Ca^2+^-containing buffer. The capillary was subsequently blocked with 10 mg ml^−1^ BSA for 2 h at room temperature. The coated capillary was connected to a 60cc syringe attached to a syringe pump (Harvard Instruments, S. Natick, MA, USA) and mounted on an inverted microscope.

Cells were harvested and the concentration was adjusted to 2×10^5^ cells ml^−1^ in DMEM without Phenol Red. Medium contained either 2 mM Ca^2+^ or EGTA (controls) at a final concentration of 5 mM. The adhesion assay was performed in low-serum medium at room temperature. Cells were drawn into the coated capillary from a reservoir, using the pump. After 1 min, the flow was stopped, and the cells were allowed to bind to the capillary surface under static conditions for 20 min at room temperature. The number of cells was then counted in a 20× field at the center of the tube where laminar flow is fully developed. After initiating flow, the number of cells remaining in the field was counted after 30 s. Subsequently, the flow was increased every 30 s, and the number of cells remaining in the field was counted at the end of each time interval.

### Statistics

The mean and standard error of the mean (s.e.m.) or standard deviation (s.d.) are reported in the text and figure legends. *P*-values were calculated from two-tailed unpaired Student's *t*-tests, using Microsoft Excel or GraphPad Prism, with *P<0.05; **P<0.005; ***P<0.0005; ****P<0.0001. When comparing three or more samples, one-way ANOVA was used. The standard errors for a set of replicate experiments were calculated using the pooled standard deviations of data from each experiment, as indicated in the text and figure legends. For confocal imaging of protein accumulation around beads in MTC measurements, the number of beads analyzed to assess protein recruitment (confocal immunofluorescence) or adaptive stiffening required for statistical significance was >100, based on prior studies and statistical power. The number of traction force measurements required for statistical significance (*n* ≥10) was based on past results and published literature. Box and whisker plots shown reveal minimum and maximum of the data, as well as inner quartiles.

## Supplementary Material

10.1242/joces.262279_sup1 Supplementary information
