# Supplementary material for: Cadherins and growth factor receptors – ligand-selective mechano-switches at cadherin junctions
Source: J Cell Sci. 2025 Feb 17;138(3):JCS262279. doi: 10.1242/jcs.262279 (PMC11883276; doi:10.1242/jcs.262279)
Supplement: Supplementary information [file joces-138-262279-s1.pdf]

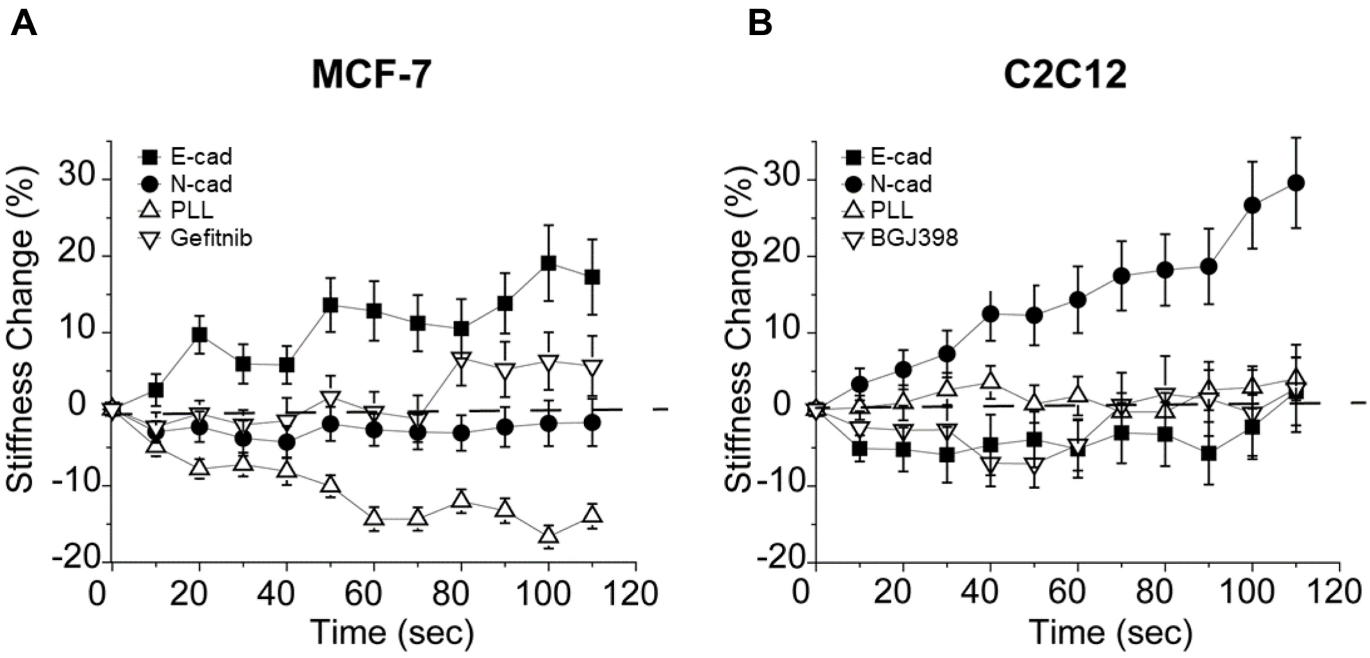

Fig. S1. Supplement to Fig. 2. (A) %Stiffness change of MCF-7 cells versus duration of loading with beads coated with E-cad (black squares), N-cad (black circles), or PLL (open triangles). Cells perturbed with E-cad beads were also treated with Gefitinib (inverted triangles). n_{beads} = 150 per condition, N_{exp}= 2. (B) %Stiffness change of C2C12cells versus duration of force loading with beads coated with E-cad (black squares), N-cad (black circles), or PLL (open triangles). Cells perturbed with E-cad beads were also treated with the FGFR inhibitor, BGJ398 (inverted triangles). n_{beads} = 150 per condition. N_{exp}= 2. Data indicate the mean ± s.d.

Journal of Cell Science • Supplementary information

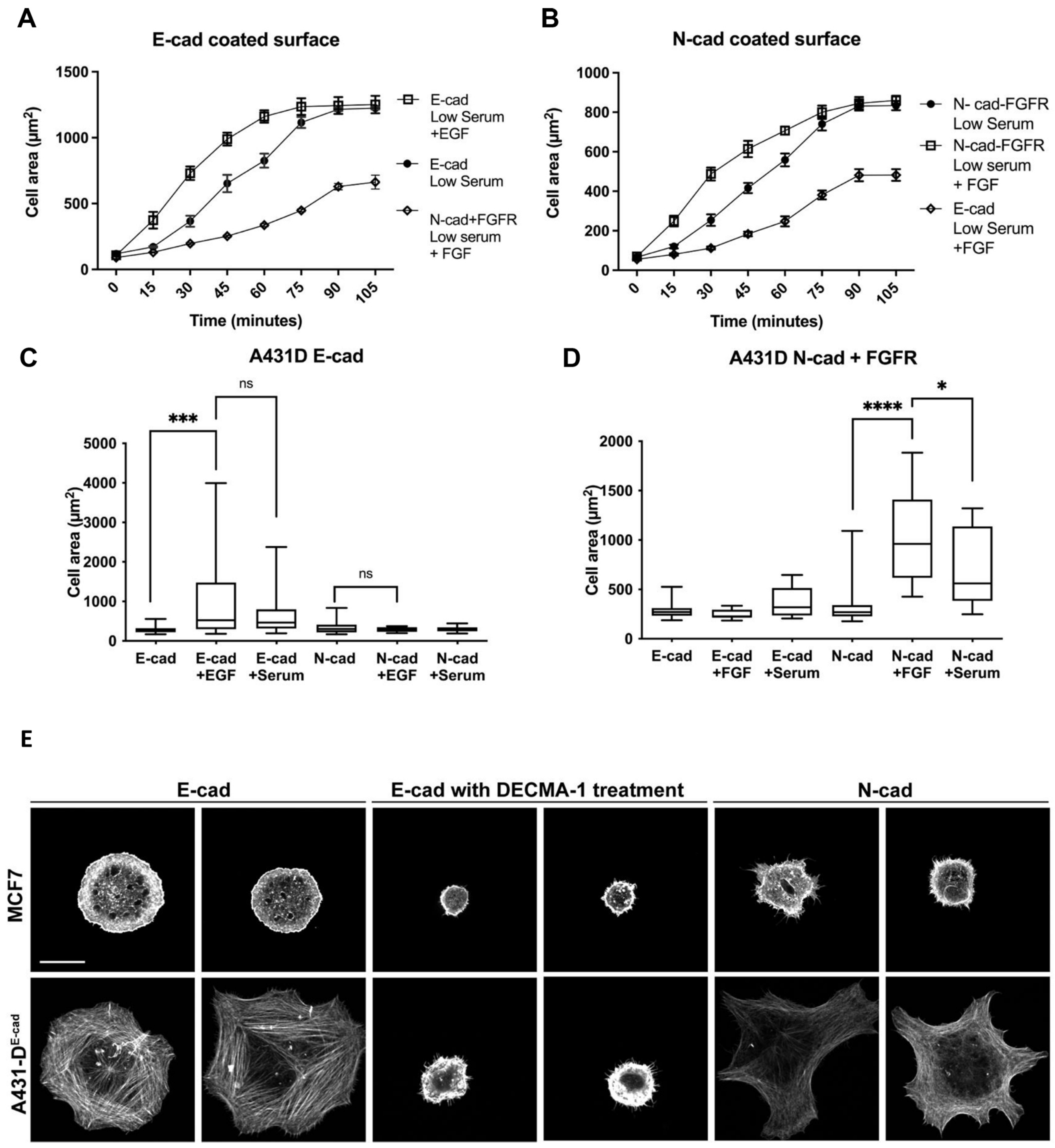

Journal of Cell Science • Supplementary information

Fig. S2. Supplement to Fig. 3. Cell spreading on cadherin substrates. (A,B) Spread area versus time after seeding cells on glass coated with E-cad or N-cad, in low serum medium. Measurements were also done ± 100 ng mL^{-1} EGF (A) or ± 100 ng mL^{-1} FGF (B). (A) Cell spreading on E-cadherin coated glass surfaces. Data show A431-$D^{E\text{-}cad}$ cells without added EGF (black circles), A431-$D^{E\text{-}cad}$ cells with added EGF (white squares), and A431-$D^{N\text{-}cad+FGFR}$ cells with added EGF (white diamonds). (B) Cell spreading on N-cad-coated glass. Data show A431-$D^{N\text{-}cad + FGFR}$ cells without added FGF (black circles), A431-$D^{N\text{-}cad + FGFR}$ cells with added FGF (white squares), and A431-$D^{E\text{-}cad}$ cells with added FGF (white diamonds). (C) Spread area (μm^2) of A431$D^{E\text{-}cad}$ cells on 40 kPa gels modified with E-cad or N-cad. Cells were cultured under conditions indicated in part C, except that 100 ng mL^{-1} EGF was used. First line under the x-axis is the gel coating and the second line indicates FGF or Serum addition (+/-). Number of cells analyzed per condition: $n_{E\text{-}cad}$ = 30, $n_{E\text{-}cad+EGF}$ = 34, $n_{E\text{-}cad+Serum}$ = 32, $n_{N\text{-}cad}$ = 21, $n_{N\text{-}cad+EGF}$ = 10, $n_{N\text{-}cad+Serum}$ = 18, N_{exp}= 3. (D) Spread area (μm^2) of A431$D^{N\text{-}cad+FGFR}$ cells on 40kPa gels modified with E-cad or N-cad. Cells were cultured in 0.5% serum medium, without or with added 100 ng mL^{-1} FGF or in 10% serum (+Serum). First line under the x-axis is the gel coating and the second line indicates FGF or Serum addition (+/-). Number of cells analyzed per condition: $n_{E\text{-}cad}$ = 16, $n_{E\text{-}cad+FGF}$ = 12, $n_{E\text{-}cad+Serum}$ = 9, $n_{N\text{-}cad}$ = 13, $n_{N\text{-}cad+FGF}$ = 13, $n_{N\text{-}cad+Serum}$ = 19, N_{exp}= 3. (E) Representative immunofluorescence images of actin at the basal planes of MCF-7 and A431$D^{E\text{-}cad}$ cells on cadherin-coated substrates. Cells were seeded in 0.5% serum containing medium. Glass substrates were coated with either E-cad-Fc or N-cad-Fc. Cells on E-cad substrates were also treated with cadherin blocking antibody DECMA-1. Two representative images are shown for each cell type and condition. The shown images were batch brightened in ImageJ, to highlight actin structures, gamma was unaffected. For representative actin images in MCF7 cells, number of images taken: $n_{E\text{-}cad}$ = 55, n_{DECMA} = 56, $n_{N\text{-}cad}$ = 55. For actin images in A431$D^{E\text{-}cad}$ cells, $n_{E\text{-}cad}$ = 110, n_{DECMA} = 70, $n_{N\text{-}cad}$ = 110. Replicates N_{exp} = 3. Scale bar: 20 microns. *p<0.05; ***p<0.0005; ns, not significant.

Journal of Cell Science • Supplementary information

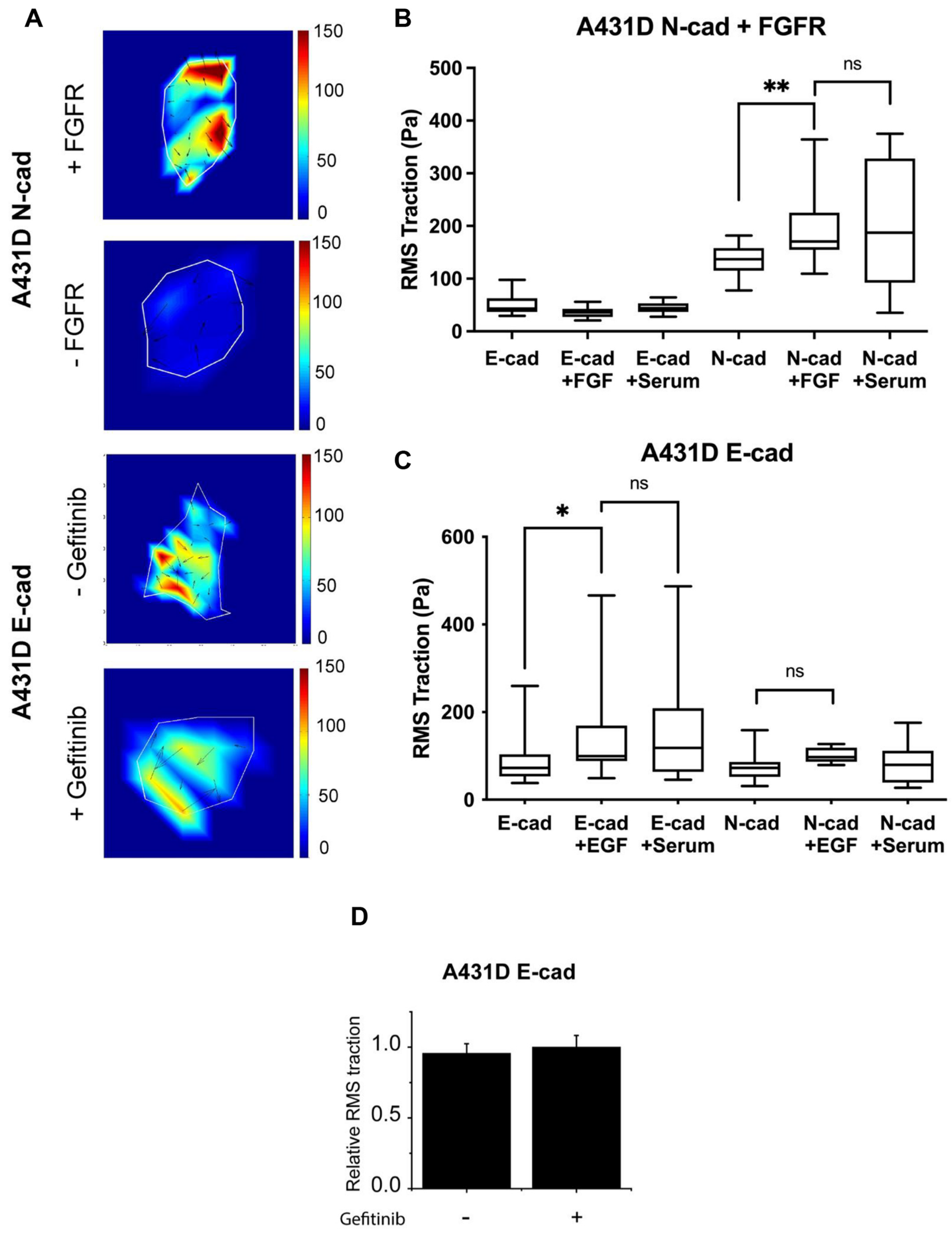

Journal of Cell Science • Supplementary information

Fig. S3. Supplement to Figure 4. (A) Representative heat maps of traction stresses generated by A431D^{N-cad} cells with (top) or without (bottom) FGFR co-expression. Representative heat maps of traction stresses generated by A431D^{E-cad} cells measured without (top) or with (bottom) Gefitinib treatment. (B) RMS traction forces of A431D$^{N-cad+FGFR}$ cells on 40kPa gels modified with E-cad or N-cad. Cells were cultured in low serum (0.5%) medium, without or with 100 ng mL^{-1} FGF or with 10% serum. In the x-axis labels, the first line indicates the cadherin substrate, and the second line indicates the addition of growth factor (+FGF) or 10% serum (+Serum). Number of cells analyzed per condition: n_{E-cad} = 16, $n_{E-cad +FGF}$ = 12, $n_{E-cad+Serum}$ = 19, n_{N-cad} = 14, $n_{N-cad+Serum}$ = 19, $n_{N-cad+FGF}$ = 13; N_{exp}= 3. (C) RMS traction forces of A431D^{E-cad} cells on gels modified with E-cad or N-cad. Cells were cultured in low serum medium (0.5%) without or with 100 ng mL^{-1} EGF. Number of cells analyzed per condition: n_{E-cad} = 37, $n_{E-cad+EGF}$ = 34, $n_{E-cad +Serum}$ = 29, n_{N-cad} = 17, $n_{N-cad+EGF}$ = 16, $n_{N-cad+Serum}$ = 16, $n_{N-cad+EGF}$ = 7; N_{exp} = 3. (D) Control for the effect of Gefitinib on integrin mediated cell tractions on collagen. Data indicate the RMS traction force generated by A431-D^{E-cad} cells on collagen-coated, 40 kPA gels in low serum medium, with or without Gefitinib treatment. *p<0.05; **p<0.005; ns, not significant.

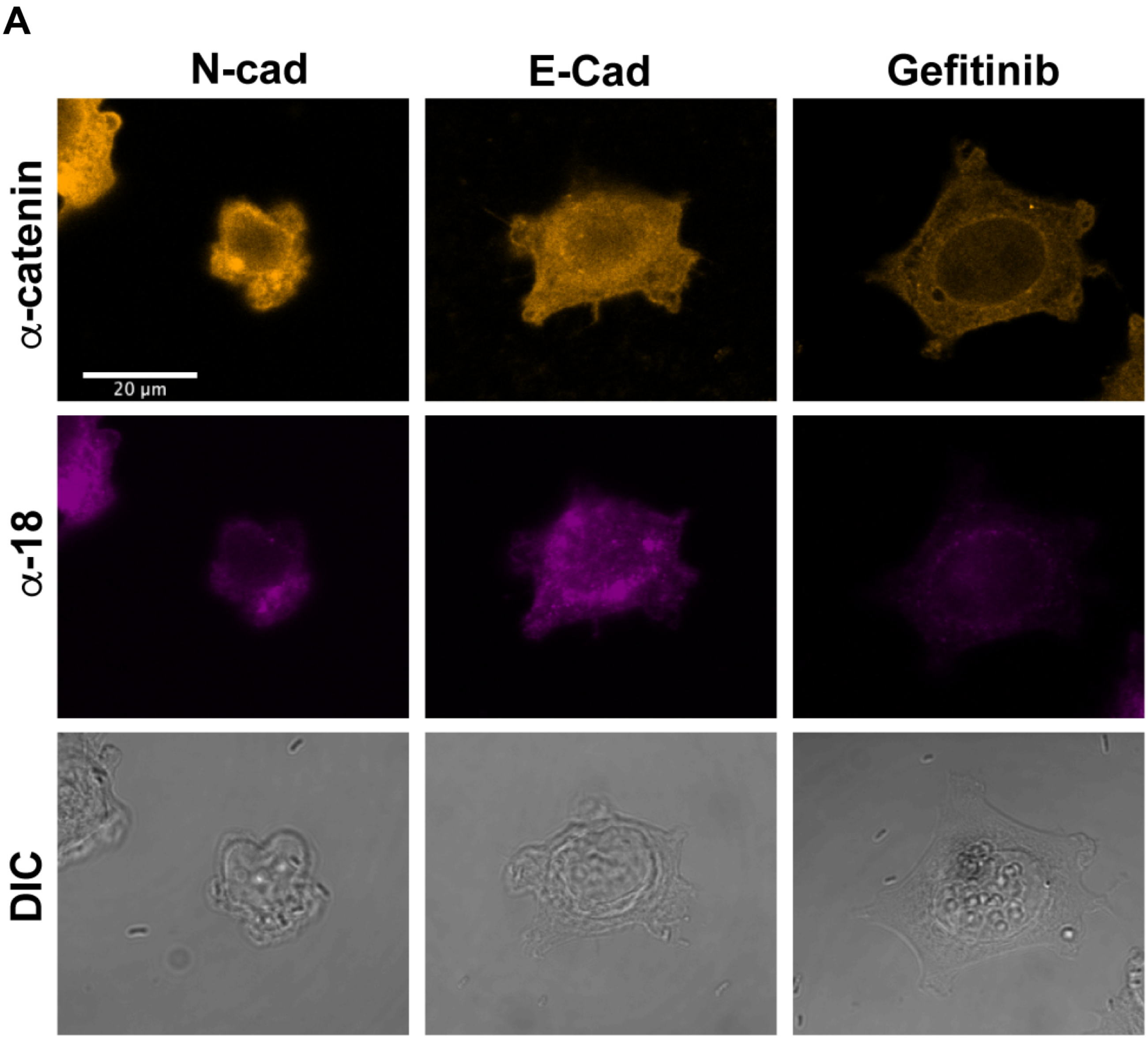

Fig. S4. Supplement to Fig. 5. Representative immunofluorescence images of α18 (top panels) and α–catenin (middle panels) staining in MCF-7 cells seeded on E-cad or N-cad coated glass substrates. Cells on E-cad substrates were also treated with Gefitinib (right column). DIC images are in the bottom row.

Journal of Cell Science • Supplementary information

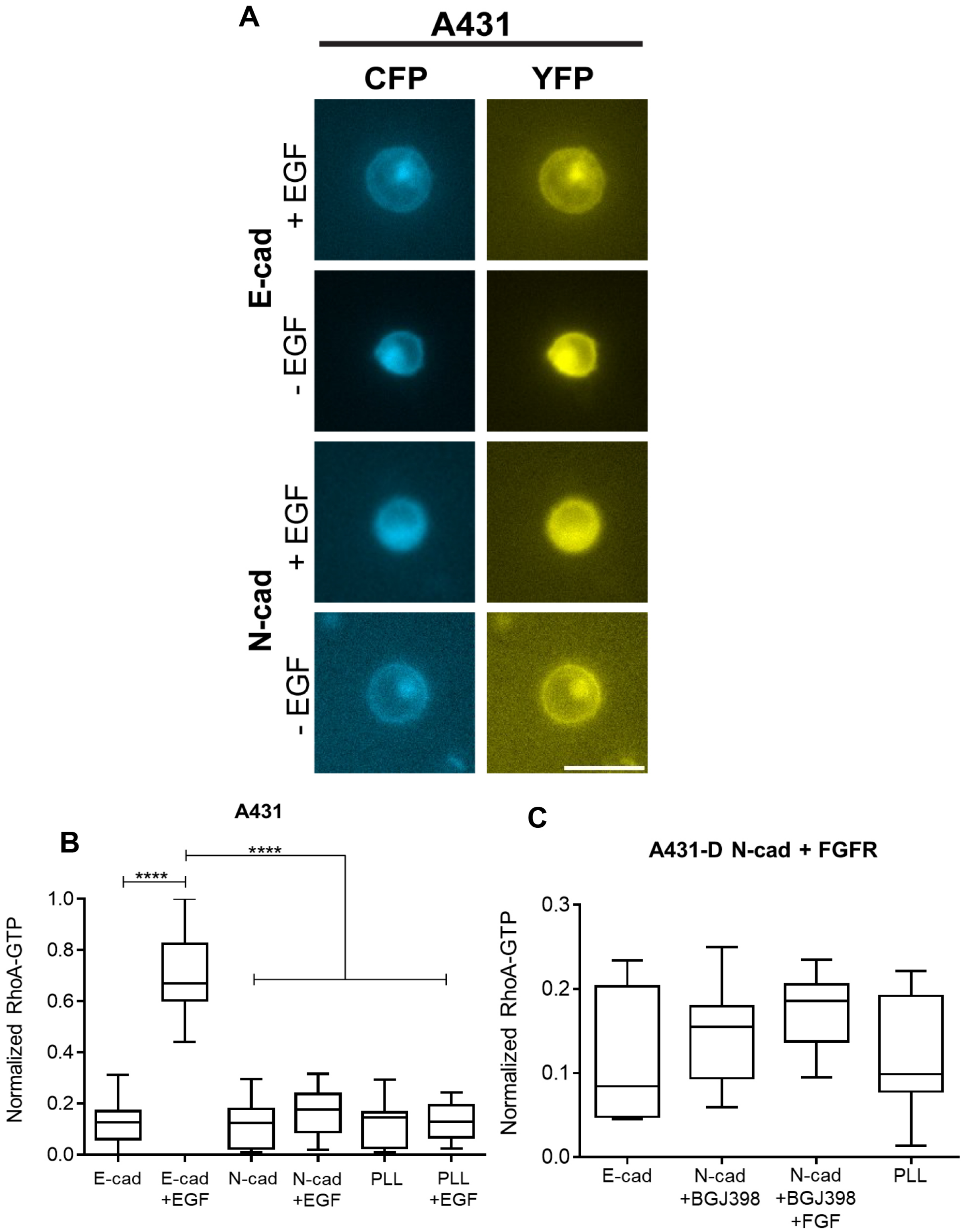

Journal of Cell Science • Supplementary information

Fig. S5. Supplement to Fig. 6. (A) G-LISA measurements of RhoA-GTP/Total RhoA levels (normalized RhoA-GTP) in A431 cells at 90 min after seeding on 40kPa polyacrylamide gels modified with E-cad, N-cad, or PLL. The first line below the x-axis indicates the cadherin substrate, and the second line indicates added 100 ng mL^{-1} of EGF. Number of gels per condition: n = 9. N_{exp}=2. **** p<0.0001. (B) G-LISA measurements RhoA-GTP/Total RhoA levels in A431-$D^{N\text{-}cad+\ FGFR}$ cells on 40kPa polyacrylamide gels coated with E-cad, N-cad, or PLL. The first line under the x-axis indicates the gel coating. The second line indicates the addition of the FGFR inhibitor (+BGJ398), and the third line indicates the addition of 100 ng mL^{-1} of FGF (+FGF). Number of gels analyzed per condition: n = 9. N_{exp}=2. ****p<0.0001.

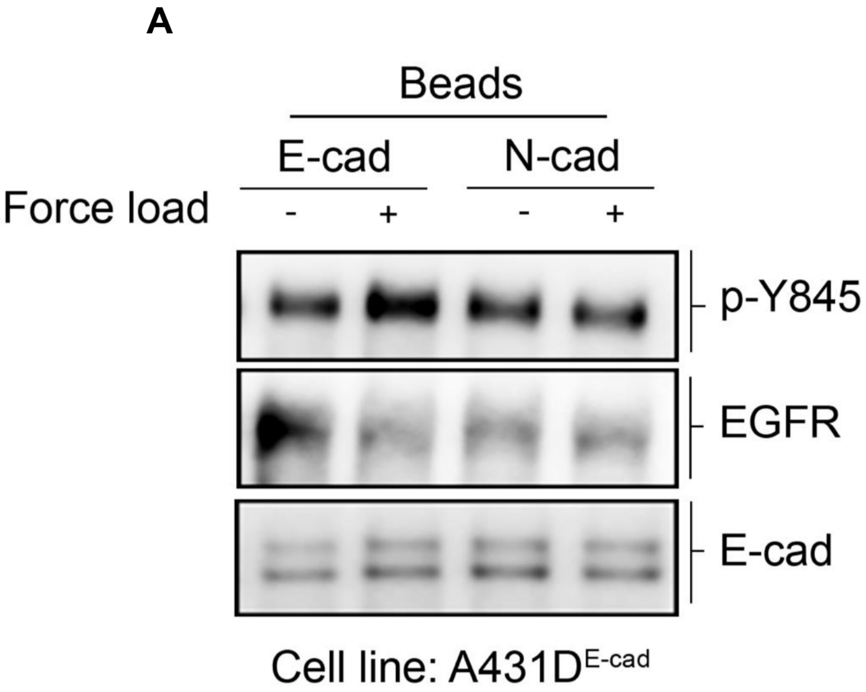

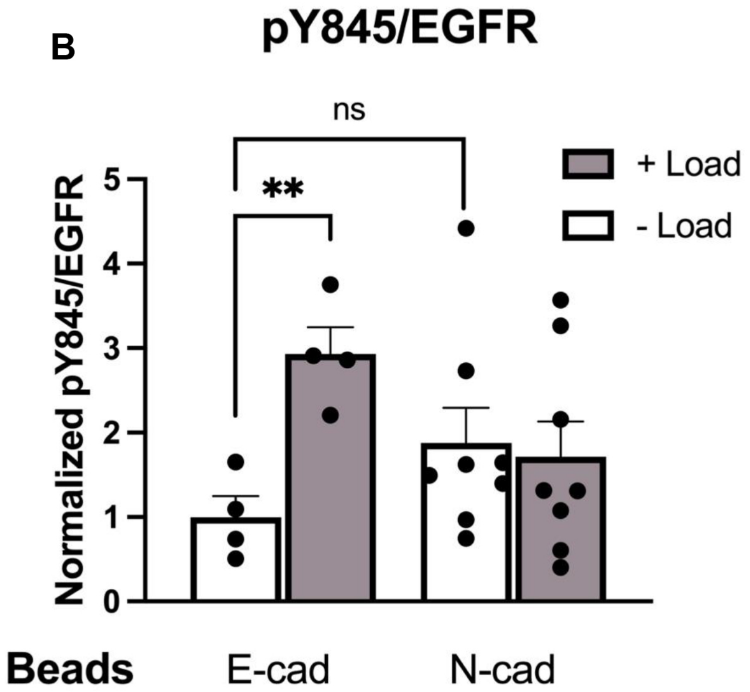

Fig. S6. Supplement to Fig. 7. (A) Representative western blot of EGFR, pY845, and E-cadherin after tugging on A431-D$^{E\text{-}cad}$ E-cadherin receptors with E-cad or N-cad modified beads. (B) Graph of the normalized ratio of pY845/EGFR in A431-D^{Ecad} cells, with (+Load) or without (-Load) bead twisting. The x-axis label indicates the bead coating. Number of blots analyzed: $n_{E\text{-}cad}$ (+/- Load) = 4; $n_{N\text{-}cad}$ (+/- Load) = 8. **p<0.005; ns, not significant.

Journal of Cell Science • Supplementary information

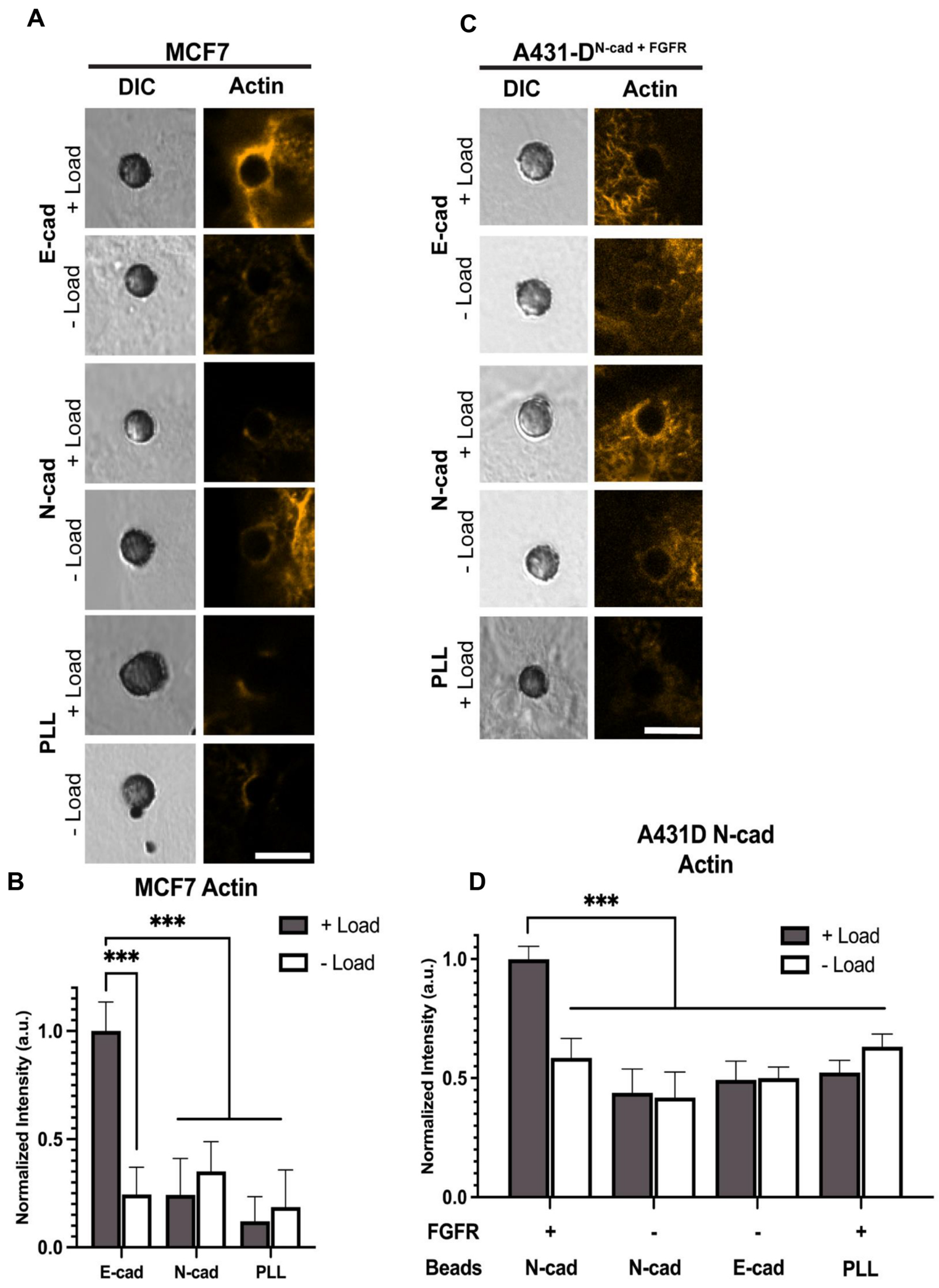

Journal of Cell Science • Supplementary information

Fig. S7. Supplement to Fig. 7. (A) DIC images of beads on MCF-7 cells and confocal immunofluorescence images of actin in regions of interest (ROI) around the bead-cell junctions, with (+Load) and without (-Load) bead twisting. Data are shown for measurements with E-cad, N-cad, or PLL coated beads. Scale bar = 8 µm. (B) Graph of the fluorescence intensities from analyses of beads in part A, normalized by the intensity at perturbed (+Load) E-cad beads. X-axis indicates the bead coating. Beads analyzed per condition: $n_{E\text{-cad}} = 45$, $n_{n\text{-cad}} = 50$, $n_{PLL} = 50$; Replicates $N_{exp} = 2$. (C) A431D$^{Ncad+FGFR}$ cells and IF images of actin at beads coated with E-cad, N-cad, or PLL, with (+Load) or without (-Load) bead twisting. (F) Graph of actin fluorescence intensities around beads on A431-D^{Ncad} cells, normalized by the intensity at perturbed (+Load) N-cad beads on A431-D^{Ncad} cells expressing FGFR (A431D$^{Ncad+FGFR}$). First line under the X-axis indicates FGFR expression (+/-) and the second line indicates the bead coating. $n_{E\text{-cad}} = 50$, $n_{n\text{-cad}} = 49$, $n_{PLL} = 50$, $n_{N\text{-cadFGFRload}} = 75$, $n_{N\text{-cadFGFRnoload}} = 30$; Replicates $N_{exp}=2$. ***p<0.0005.

Journal of Cell Science • Supplementary information

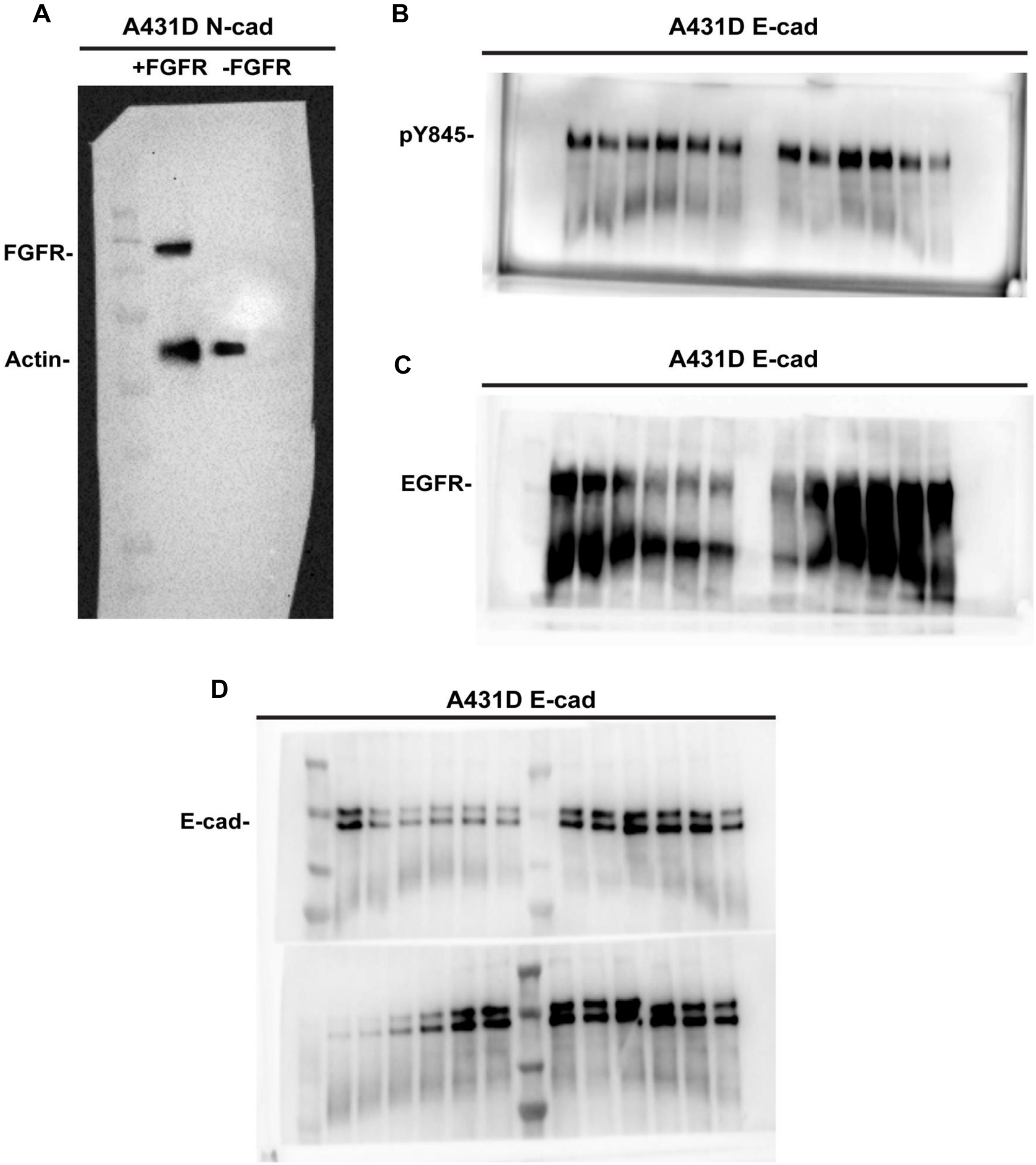

Fig. S8. Supplement to Figs. 2 and S6. Representative western blots (A) Confirmation of FGFR transfection (upper left band at 140 kDa) and actin (42 kDa) in A431D$^{N\text{-}cad+FGFR}$ cells. (B) EGFR activation at pY845 in A431D$^{E\text{-}cad\text{-}YFP}$ cells in response to tugging force by homotypic ligation, but not with heterotypic interaction. (C) EGFR blotting of A431D$^{E\text{-}cad\text{-}YFP}$ cells. (D) E-cadherin blotting in A431D$^{E\text{-}cad\text{-}YFP}$ cells.

Journal of Cell Science • Supplementary information
